# CGRP–RAMP1/CALCRL Signaling Drives TGF-β2-Mediated Immune Evasion and Pancreatic Cancer Progression

**DOI:** 10.3390/ijms27209108

**Published:** 2026-10-14

**Authors:** Biyu Zhang, Yaxin Li, Simiao Fan, Jiamei Zhang, Yu Zhang, Wen Wang, Liang Luo, Wanying Xiao, Xinlei Xie, Xiaoxiao Jian, Qiuying Han, Shaoyi Huang, Min Wu, Zengqing Song, Yuqi Wen, Haiqing Tu, Sen Li, Huaibin Hu

**Affiliations:** 1 Nanhu Laboratory, State Key Laboratory of Biomedical Analysis (SKLBA, Formerly Known as National Center of Biomedical Analysis), Beijing 100039, China; byzhang@xmail.ncba.ac.cn (B.Z.); lyxyd2019@163.com (Y.L.);; 2 School of Basic Medical Sciences, Fudan University, Shanghai 200032, China

**Keywords:** PDAC, mechanism, T cell, CGRP, RAMP1, CALCRL, TGF-β

## Abstract

Pancreatic ductal adenocarcinoma (PDAC) is characterized by extensive neural remodeling and a profoundly immunosuppressive tumor microenvironment, both of which contribute to disease progression and therapeutic resistance. However, whether nociceptor-derived signals directly reprogram PDAC cells to promote immune evasion remains poorly understood. Here, we identify a previously unrecognized tumor-intrinsic neuroimmune mechanism driven by calcitonin gene-related peptide (CGRP) signaling through the receptor complex RAMP1/CALCRL on PDAC cells. CRISPR/Cas9-mediated deletion of *Ramp1* or *Calcrl* did not alter PDAC cell proliferation in vitro but markedly suppressed tumor growth and prolonged survival in immunocompetent mice. These antitumor effects were lost in immunodeficient hosts, indicating that the pro-tumorigenic function of CGRP signaling depends on an intact host immune system. Consistently, tumor cell-specific loss of *Ramp1* or *Calcrl* remodeled the tumor immune microenvironment, enhancing CD4^+^ and CD8^+^ T-cell effector activity. Mechanistically, CGRP stimulation promoted TGF-β2 expression and secretion by PDAC cells in a RAMP1-dependent manner. Tumor-derived TGF-β2 subsequently acted in a paracrine fashion to suppress CD8^+^ T-cell activation and cytotoxic function, whereas genetic disruption of RAMP1 or pharmacological inhibition of TGF-β signaling relieved this immunosuppression. Together, these findings define a CGRP–RAMP1/CALCRL–TGF-β2 signaling axis through which nociceptive input is converted into tumor-driven adaptive immune evasion in PDAC. This pathway reveals a previously underappreciated mechanism linking neural signaling to PDAC progression and identifies CGRP receptor signaling as a potential therapeutic vulnerability for restoring antitumor immunity and improving the responsiveness of PDAC to immunotherapy.

## 1. Introduction

Pancreatic ductal adenocarcinoma (PDAC) is one of the most lethal solid tumors [1] characterized by perineural invasion (PNI) [2,3,4,5]. Unlike the localized perivascular and interlobular distribution in the normal pancreas, the PDAC parenchyma undergoes extensive neural remodeling, characterized by significantly increased nerve density and diffuse infiltration [6,7]. Specifically, calcitonin gene-related peptide-positive (CGRP^+^) nociceptive, tyrosine hydroxylase-positive (TH^+^) sympathetic [8], and vesicular acetylcholine transporter-positive (VAChT^+^) parasympathetic fibers densely infiltrate and interlace throughout the tumor bed [6], collectively sculpting a topologically rewired neural landscape. This structurally rewired neural terrain is accompanied by a functionally diverse repertoire of neural signals, among which several distinct neurotransmitter/neuropeptide axes have been implicated in PDAC progression [7,9].

Among these, parasympathetic nerve-derived cholinergic signals have been shown to directly suppress PDAC cell proliferation and diminish stemness, thereby attenuating tumor progression [5,10,11,12]. In contrast, catecholamines derived from sympathetic nerves within PDAC directly act on ADRB2 expressed on PDAC cells to promote tumor progression [5,12,13], and intestinal neuron-derived vasoactive intestinal peptide (VIP) engages the VPAC2 receptor on PDAC cells to accelerate tumor progression [14,15].

PDAC is also heavily innervated by nociceptive nerves [16,17,18]. Nociceptor-derived neurotransmitters (glutamate and aspartate) and neuropeptides (calcitonin gene-related peptide (CGRP) [19], substance P (SP) [16]) broadly regulate the PDAC microenvironment. It has been shown that CGRP acts on the RAMP1/CALCRL complex on cancer-associated fibroblasts (CAFs) to reduce IL-15 secretion, thereby impairing NK cell function and promoting pancreatic cancer progression [19]. Additionally, substance P enhances the invasiveness of human pancreatic cancer cells via NK-1R (the receptor for substance P) [20,21]. Notably, cytotoxic CD8^+^ T cell exclusion represents a key mechanism underlying resistance to ICB therapy in PDAC patients [2,11,22,23,24], and CGRP directly promotes the exhaustion of tumor-infiltrating CD8^+^ T cells in B16-F10 tumors via RAMP1 [25]. However, whether nociceptive nerves directly act on PDAC tumor cells via these neural signals to influence tumor progression remains unclear [26,27,28].

Here, we demonstrate that PDAC tumor cells abundantly express RAMP1 and CALCRL, the receptors for CGRP [29,30]. Although genomic ablation of RAMP1 or CALCRL did not affect PDAC cell growth in vitro, their deficiency significantly restricted subcutaneous tumor growth in vivo in an immune-dependent manner. Flow cytometric analysis of the immune microenvironment revealed that CGRP receptor-deficient tumors exhibited enriched infiltration of effector T cells. Mechanistically, the CGRP–RAMP1/CALCRL axis promoted TGF-β2 secretion by PDAC cells, which subsequently suppressed CD8^+^ T cell effector function. Collectively, our findings unveil a novel mechanism by which CGRP orchestrates T cell anti-tumor immunity via tumor cell-expressed CALCRL/RAMP1, offering new insights for PDAC therapy.

## 2. Results

### 2.1. RAMP1 and CALCRL Are Highly Expressed on Pancreatic Cancer Cells

**Figure 1:**
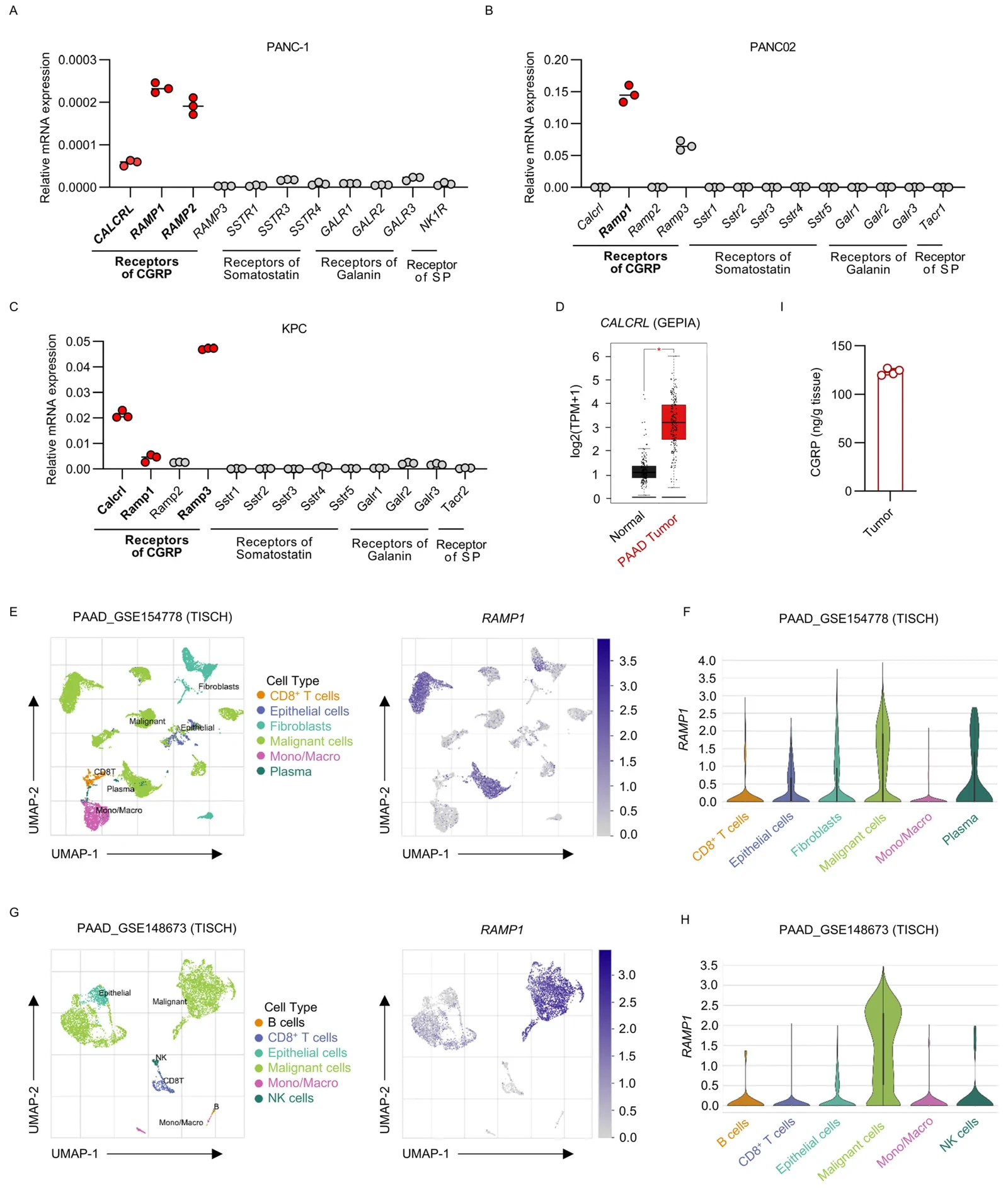
RAMP1 and CALCRL expression in human and mouse pancreatic cancer cells. (**A**–**C**) The relative mRNA expression levels of genes encoding receptors for nociceptive neurotransmitters and neuropeptides in human (**A**) and mouse (**B**,**C**) pancreatic cancer cell lines. Data were obtained from three independent biological replicates using cells from different passages, with cDNA independently prepared for each biological replicate (*n* = 3 independent biological replicates). (**D**) *CALCRL* expression in pancreatic adenocarcinoma (PAAD) and corresponding normal tissues obtained from the Gene Expression Profiling Interactive Analysis (GEPIA 2.0). The asterisk indicates a statistically significant difference between the indicated groups. (**E**–**H**) Based on the single-cell sequencing data from the TISCH database datasets PAAD_GSE154778 [36] and PAAD_GSE148673 [37], *RAMP1* is abundantly expressed in the tumor cells of human pancreatic cancer. (**I**) CGRP concentrations in PANC02 subcutaneous tumors from C57BL/6J mice, as determined by ELISA (*n* = 4 mice; each mouse represents an independent biological replicate).

To investigate whether nociceptor-derived neurotransmitters or neuropeptides can directly act on pancreatic cancer cells, we first quantified the expression of their receptors in human (PANC-1) and murine (PANC02 and KPC) pancreatic cancer cell lines, including CGRP [31], Somatostatin [32], Galanin [33] and SP [32]. Among the multiple receptors tested, RAMP1 and CALCRL, the receptors of CGRP showed relatively high expression across these cell lines (Figure 1A–C). To validate their expression in clinical cohorts, we interrogated the TCGA database via GEPIA 2.0 [34] and retrieved the expression plots shown in this study. The results revealed that *CALCRL* transcripts were significantly upregulated in human pancreatic adenocarcinoma (PAAD) tissues compared to adjacent normal tissues [35] (Figure 1D). To further resolve the cell-type-specific distribution of these receptors within the tumor microenvironment, we analyzed two independent single-cell RNA-seq datasets (PAAD_GSE154778 [36] and PAAD_GSE148673 [37]) from the TISCH 2.0 database [38,39]. UMAP clustering and violin plot analyses showed that *RAMP1* was prominently distributed within the malignant tumor cell clusters [36,40] (Figure 1E–H). Given that an enriched presence of CGRP has been reported within human PDAC tissues [19], we next evaluated the local concentration of CGRP in vivo. Analysis of a murine PANC02 subcutaneous tumor model demonstrated that CGRP protein levels within the PDAC tumor mass exceeded 100 ng per gram of tissue (Figure 1I). Collectively, we found that both human and mouse pancreatic cancer cells express RAMP1 and CALCRL, the receptors for the nociceptive neuropeptide CGRP.

### 2.2. Ramp1/Calcrl Deficiency Does Not Affect PDAC Cell Proliferation In Vitro

**Figure 2:**
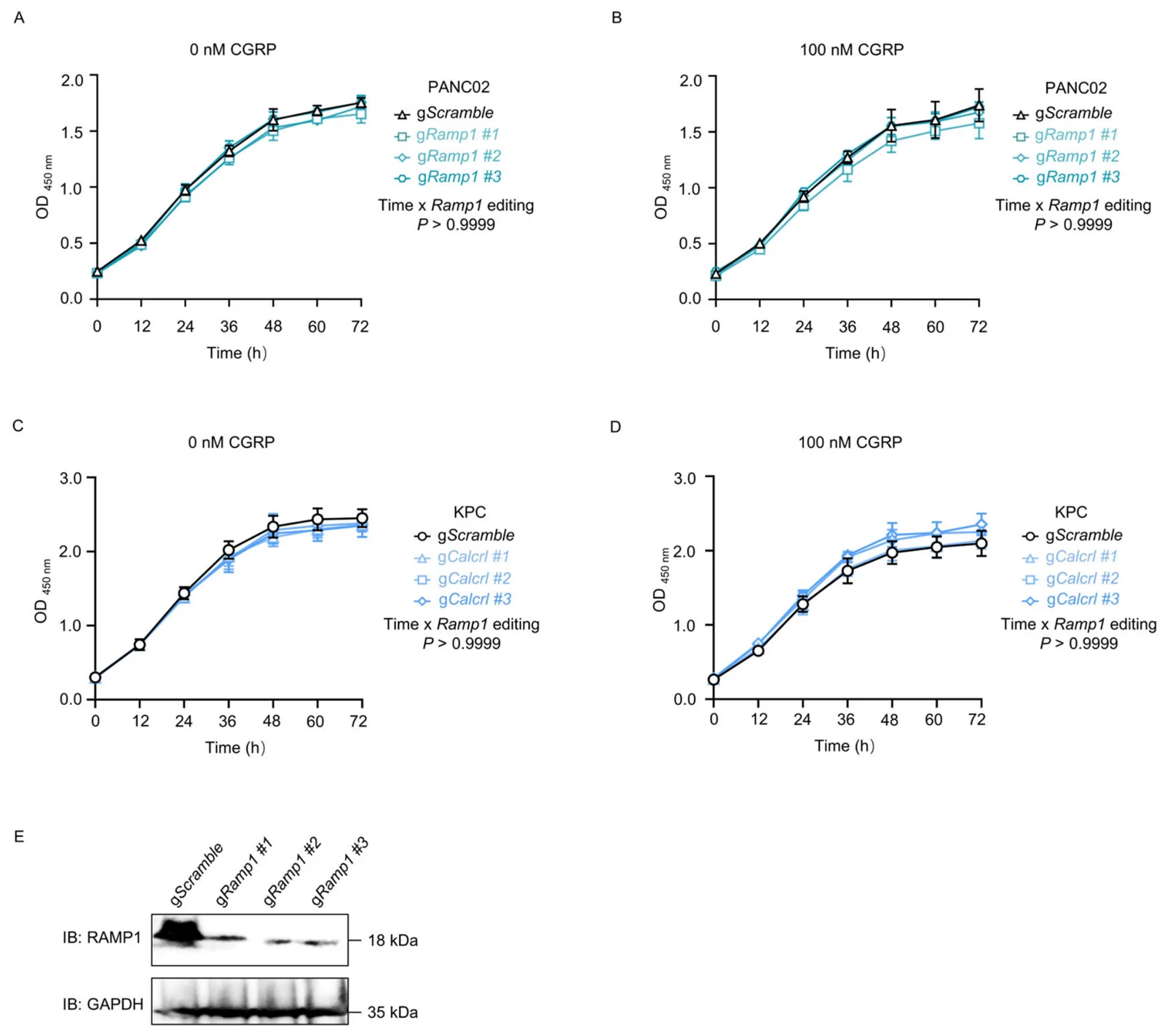
*Ramp1* and *Calcrl* deficiencies have no effect on the in vitro growth of PANC02 and KPC cells with or without CGRP. (**A**) CCK-8 assay for PANC02 cell growth without CGRP. (**B**) CCK-8 assay for PANC02 cell growth with 100 nM CGRP. (**C**) CCK-8 assay for KPC cell growth in vitro without CGRP. (**D**) CCK-8 assay for KPC cell growth in vitro with 100 nM CGRP. Data are presented as mean ± SD from four independent biological replicates using cells from different passages (*n* = 4). Cell growth curves in (**A**–**D**) were analyzed using two-way repeated-measures ANOVA with the Geisser–Greenhouse correction, followed by Dunnett’s multiple-comparisons test. (**E**) Western blot analysis of RAMP1 protein expression in g*Scramble* and g*Ramp*1 #1, #2, and #3 PANC02 cells.

CGRP signaling strictly requires the heteromeric CALCRL-RAMP1 complex; neither component alone is sufficient. CALCRL requires RAMP1 as an obligate partner for CGRP signaling, enabling not only ligand recognition and membrane localization, but also proper glycosylation and trafficking during receptor maturation. To investigate the functional role of the CALCRL-RAMP1 receptor complex in pancreatic cancer cells, we performed individual knockout of either RAMP1 or CALCRL in pancreatic cancer cell lines and assessed the subsequent effects on cell viability. First, we generated *Ramp1*-deficient PANC02 cells using a CRISPR/Cas9 Lenti-V2 lentiviral system (Figure S2A). The target sequences and corresponding genomic locations of the sgRNAs are provided in Table S1. The knockout effect was confirmed by DNA sequencing (Figure S2B,C). We then performed CCK-8 cell viability assays to determine whether CGRP signaling directly modulates cell-autonomous growth kinetics. Notably, *Ramp1*-deficient PANC02 cells exhibited identical proliferation rates compared to their control counterparts, with or without CGRP (Figure 2A,B). Similarly, we generated *Calcrl*-deficient KPC cells by CRISPR/Cas9 RNP electroporation system (Figure S2D), with the sgRNA target sequences and corresponding genomic locations listed in Table S2, with successful targeting confirmed by DNA sequencing (Figure S2E,F). Consistent with our observation in the PANC02 cells, the genetic ablation of *Calcrl* yielded no detectable differences in KPC cell growth or viability in vitro (Figure 2C,D). Collectively, these data indicate that genetic deletion of *Ramp1* or *Calcrl* in pancreatic cancer cells does not impair cell-autonomous proliferation in vitro. Western blot analysis further showed markedly reduced RAMP1 protein abundance in the g*Ramp1* #1, #2, and #3 PANC02 clones (Figure 2E). For *Calcrl*-edited KPC clones, direct protein-level confirmation was limited by the lack of a sufficiently specific CALCRL antibody; clone validation therefore relied on genomic PCR and Sanger sequencing. mRNA abundance was not used as the primary knockout-efficiency criterion because frameshifted transcripts may be variably affected by nonsense-mediated decay.

### 2.3. Ramp1/Calcrl Deficiency in Pancreatic Cancer Cells Inhibited Tumor Growth in Immunocompetent Mice

**Figure 3:**
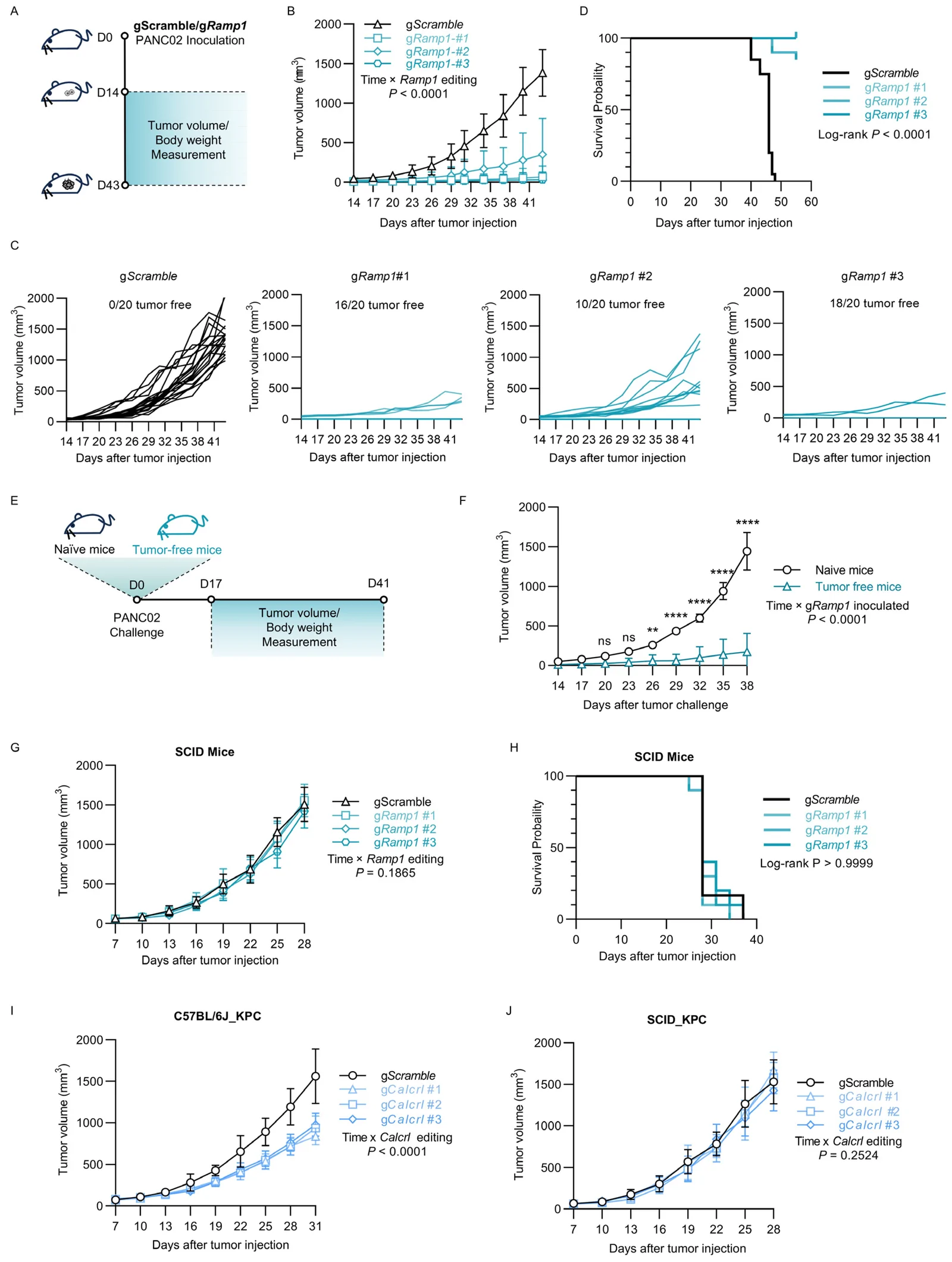
*Ramp1* and *Calcrl* deficiencies inhibit subcutaneous tumor growth in an immune-dependent manner. (**A**–**D**) PANC02 cells were inoculated subcutaneously in C57BL/6J mice (*n* = 20 per group) (**A**), and the tumor volume was measured from day 14 after inoculation (**B**,**C**). (**D**) Mice with a tumor volume reaching 1500~1700 mm^3^ reached the humane endpoint. These mice were euthanized, and their overall survival time was recorded. (**E**,**F**) Tumor-free mice (i.e., those subcutaneously inoculated with *Ramp1*-deficient PANC02 that never developed tumors) and age-matched naïve C57BL/6J mice were re-challenged with wild-type PANC02. *n* = 5 per group. ns, not significant; ** *p* < 0.01; **** *p* < 0.0001. (**G**,**H**) PANC02 cells were inoculated subcutaneously in SCID mice (*n* = 10 per group); the tumor volume was measured from day 7 after inoculation (**G**), and their overall survival time was recorded (**H**). (**I**) KPC cells were inoculated subcutaneously in C57BL/6J mice, and the tumor volume was measured from day 7 after inoculation; *n* = 10 per group. (**J**) KPC cells were inoculated subcutaneously in SCID mice, and the tumor volume was measured from day 7 after inoculation; *n* = 10 per group. Tumor growth curves in (**B**,**F**,**G**,**I**,**J**) were analyzed using two-way repeated-measures ANOVA with the Geisser–Greenhouse correction, followed by Dunnett’s multiple-comparisons test for comparisons between the indicated experimental groups and their corresponding control groups at individual time points. The *p* values displayed in the tumor growth graphs represent the time × experimental group interaction from the two-way repeated-measures ANOVA. Survival curves in (**D**,**H**) were estimated using the Kaplan–Meier method and compared using the log-rank (Mantel–Cox) test. All statistical analyses were performed using GraphPad Prism. Exact *p* values are shown in the graphs. Longitudinal measurements were initiated when tumors had generally reached approximately 30–80 mm^3^; this corresponded to day 14 for the PANC02 C57BL/6J cohort and approximately day 7 for the other tumor-growth cohorts. Each mouse was treated as an independent biological replicate.

To evaluate the functional significance of RAMP1 and CALCRL during PDAC progression in vivo, we first subcutaneously inoculated wild-type (g*Scramble*) or *Ramp1*-deficient (g*Ramp1*) PANC02 cells into C57BL/6J mice (Figure 3A). Notably, all *Ramp1*-deficient PANC02 cell lines derived from three different sgRNAs exhibited significantly impaired tumor growth compared to WT controls (Figure 3B,C). A substantial proportion of mice inoculated with *Ramp1*-deficient PANC02 cells remained tumor-free (Figure 3C and Figure S1A), which translated into a significantly prolonged tumor endpoint-free survival (Figure 3D). This impaired growth phenotype was highly reproducible in independent experimental cohorts (Figure S1B).

Moreover, to determine whether the rejection of *Ramp1*-deficient PDAC cells elicited durable anti-tumor immune memory, the surviving tumor-free mice were re-challenged with WT PANC02 cells (Figure 3E). Interestingly, these mice robustly rejected the secondary challenge of WT PANC02 cells (Figure 3F), suggesting that initial exposure to *Ramp1*-deficient tumor cells establishes protective adaptive immunity. To test whether this protective effect is immune-dependent, we inoculated WT or *Ramp1*-deficient PANC02 cells into immunodeficient SCID mice. In striking contrast to the immunocompetent setting, *Ramp1* deficiency failed to alter tumor growth or survival in SCID hosts (Figure 3G,H and Figure S1C).

Similarly, *Calcrl*-deficient KPC cell lines showed markedly reduced tumor growth and prolonged survival in immunocompetent C57BL/6J mice compared to WT controls (Figure 3I and Figure S1D,E), whereas this tumor-suppressive effect was abrogated in SCID recipients (Figure 3J and Figure S1F). Taken together, these data demonstrate that tumor cell-expressed RAMP1 and CALCRL promote pancreatic cancer progression in a host immune-dependent manner.

### 2.4. Ramp1/Calcrl Deficiency in Pancreatic Cancer Cells Reprograms Tumor Immune Microenvironment

**Figure 4:**
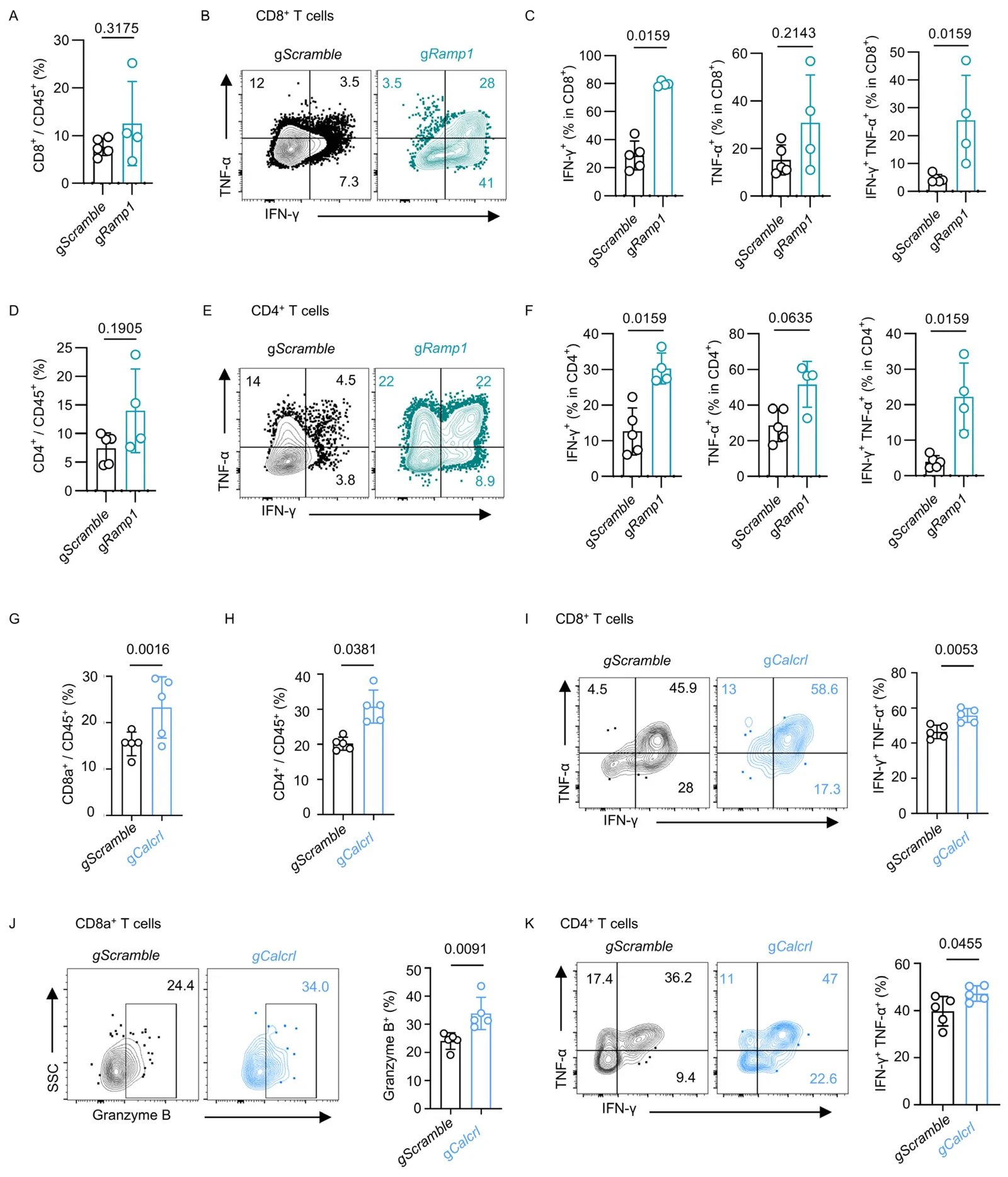
Flow cytometric analysis of the tumor immune microenvironment (TIME) in PANC02 and KPC tumors in C57BL/6J mice. (**A**–**F**) Comparative analysis of tumor-infiltrating T cells in PANC02 tumors (control group, *n* = 5; experimental group, *n* = 4). One sample from the experimental group was excluded from the analyses shown in (**A**–**F**) because the number of acquired T-cell events was insufficient for reliable downstream cytokine subset analysis. Percentages of CD8^+^ T cells in CD45^+^ cells (**A**). Percentages of TNF-α^+^ and/or IFN-γ^+^ in CD8^+^ T cells (**B**,**C**). Percentages of CD4^+^ T cells in CD45^+^ cells (**D**). Percentages of TNF-α^+^ and/or IFN-γ^+^ in CD4^+^ T cells (**E**,**F**). (**G**–**K**) Comparative analysis of T cells infiltrated in KPC tumors in C57BL/6J mice, *n* = 5 per group. Percentages of CD8^+^ T cells (**G**) and CD4^+^ T cells (**H**) in CD45^+^ cells. Percentages of TNF-α^+^ and IFN-γ^+^ cells in CD8^+^ T cells (**I**) and CD4^+^ T cells (**K**). Percentages of Granzyme-B^+^ cells in CD8^+^ T cells (**J**). Data are presented as mean ± SD. For the PANC02 analysis, each point represents one independently analyzed biological sample. When 2 very small *Ramp1*-deficient tumors were pooled to obtain sufficient T cells, the resulting pool was treated as one analytical sample rather than as multiple independent mice. Comparisons between two independent groups were performed using the two-tailed Mann–Whitney U test. Exact *p* values are shown in the graphs.

Given the immune-dependent tumor rejection in vivo, we next sought to investigate how the RAMP1/CALCRL signaling influences tumor immunity in PDAC. To this end, we characterized the composition of tumor-infiltrating immune cells using flow cytometry. The antibodies used, together with their clone numbers and sources, are listed in Table S3. In *Ramp1*-deficient PANC02 tumors, CD4^+^ and CD8^+^ T cell infiltration showed an increasing trend without statistical significance (Figure 4A,D). Moreover, the cytotoxicity of CD8^+^ T cells was notably enhanced, marked by a significant increase in the proportion of IFN-γ^+^, TNF-α^+^ and IFN-γ^+^ TNF-α^+^ double-positive cells (Figure 4B,C). Consistently, CD4^+^ T cells also exhibited significantly enhanced effector cytokine production, with elevated IFN-γ^+^, TNF-α^+^ and IFN-γ^+^ TNF-α^+^ proportions (Figure 4E,F), while regulatory T cell (Tregs) proportions remained unchanged (Figure S3D). Interestingly, the mean fluorescence intensity (MFI) of the exhaustion marker PD-1 on CD8^+^ T cells was significantly downregulated in *Ramp1*-deficient PANC02 tumors (Figure S3C), despite the total numbers of exhausted T cells and terminally exhausted T cells remaining unchanged (Figure S3A,B).

In *Calcrl*-deficient KPC tumors, we observed significantly increased infiltration of cytotoxic lymphocytes, including CD8^+^ T cells and CD4^+^ T cells (Figure 4G,H). Furthermore, similar to the *Ramp1*-deficient tumor model, *Calcrl* deficiency enhanced the cytotoxicity of both CD8^+^ and CD4^+^ T cells (Figure 4I–K). In conclusion, these data demonstrate that genetic deletion of *Ramp1* or *Calcrl* in pancreatic cancer cells reprograms the tumor microenvironment and boosts T cell-mediated antitumor immunity.

### 2.5. Ramp1 Deficiency in PDAC Cells Abrogates TGF-β Signals on Tumor Cells

**Figure 5:**
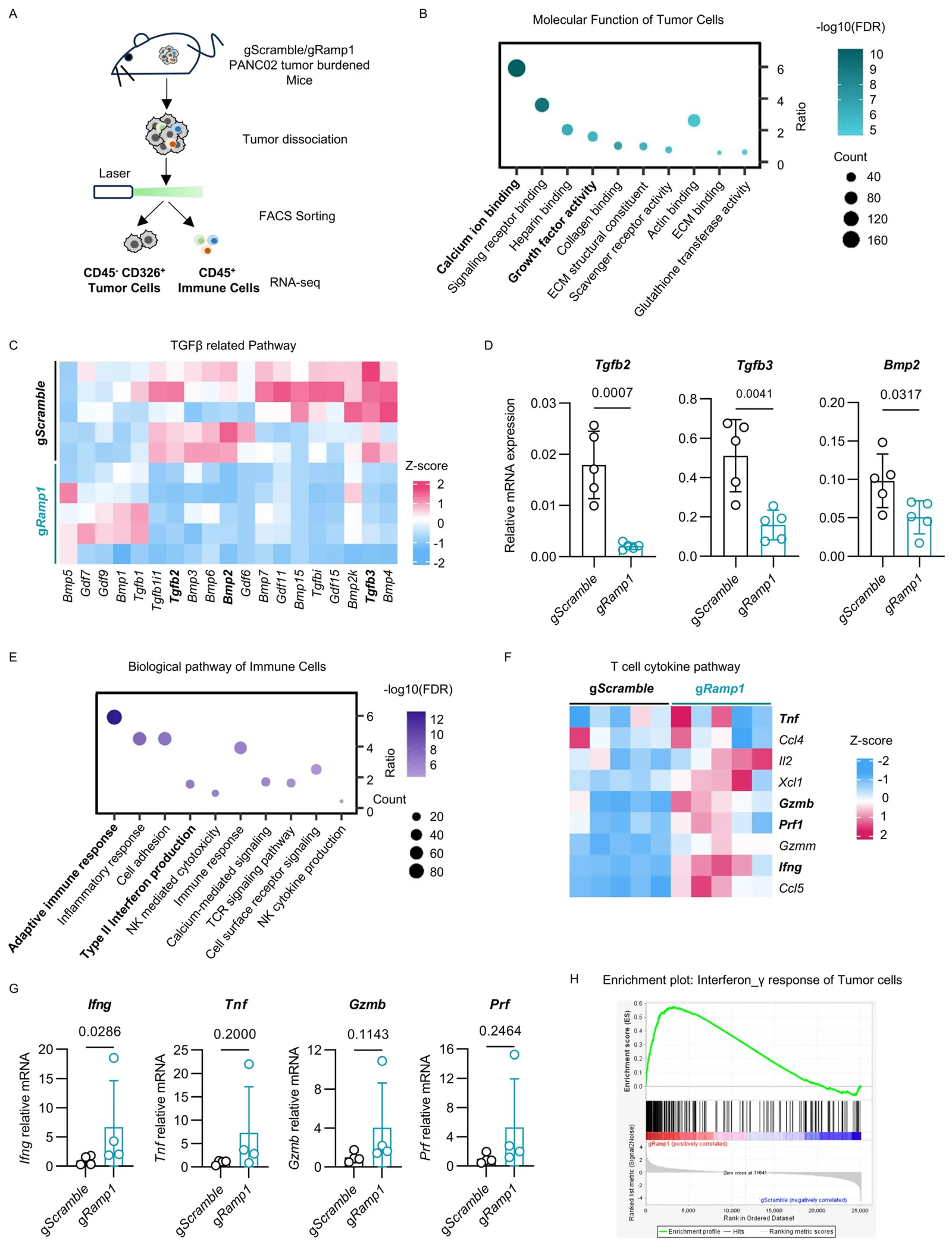
RNA sequencing analysis of tumor and immune cell components from PANC02 tumors with or without *Ramp1* deficiency. (**A**) The flow cytometry sorting strategy of tumor cells (CD45^−^ CD326^+^) and immune cells (CD45^+^) in PANC02 tumors, *n* = 5 per group. (**B**) GO analysis of molecular functions of tumor cells (CD45^−^ CD326^+^). (**C**) Hierarchical clustering heatmap of gene expression in the TGF-β/BMP signaling pathways in tumor cells (CD45^−^ CD326^+^), generated using the Hiplot platform. Rows: genes; columns: samples; color scale: Z-score-normalized expression (blue, low; red, high). (**D**) qPCR validation of the relative expression of *Tgfb1*, *Tgfb2*, *Tgfb3*, and *Bmp2* in the remaining independently collected samples sorted from PANC02 tumors, *n* = 5 per group. (**E**) GO analysis of biological pathways of immune cells (CD45^+^). (**F**) Hierarchical clustering heatmap of gene expression of T cell-related cytokines and cytotoxic genes in immune cells (CD45^+^), generated using the Hiplot platform. Rows: genes; columns: samples; color scale: Z-score-normalized expression (blue, low; red, high). (**G**) qPCR validation of representative T-cell effector and cytokine-related genes identified by RNA-seq. CD45^+^ tumor-infiltrating immune cells were isolated from g*Scramble* and g*Ramp1* PANC02 tumors, and the relative mRNA expression levels of *Ifng*, *Tnf*, *Gzmb*, and *Prf1* were determined by qPCR (*n* = 4 mice per group). Each point represents one mouse (independent biological replicate). Data are presented as mean ± SD. Comparisons between the gScramble and gRamp1 groups were performed using the two-tailed Mann–Whitney U test. Exact *p* values are shown in the graphs. (**H**) GSEA of *Ramp1*-deficient versus gScramble PANC02 tumor cells (CD45^−^CD326^+^), showing the interferon-γ response gene set. In (H), the green line represents the enrichment score profile, the black vertical bars indicate the positions of genes in the gene set within the ranked gene list, and the red-to-blue color gradient represents the ranked-list metric, with red and blue indicating positive and negative correlations, respectively. The normalized enrichment score (NES) was −2.4174209; Nominal *p* value < 0.001; FDR q < 0.25. Comparisons in (**D**) were performed using an unpaired *t* test; exact *p* values are shown in the graphs. GO enrichment analyses in (**B**,**E**) were considered statistically significant at an adjusted *p* value < 0.05, with multiple-testing correction performed using the Benjamini–Hochberg method.

Based on the observation that *Ramp1* or *Calcrl* deficiency significantly alters the tumor immune microenvironment, we sought to elucidate the intrinsic molecular mechanisms by which PDAC cells communicate with infiltrating immune cells within the tumor. We sorted tumor cells (CD45^−^ CD326^+^) and immune cells (CD45^+^) from WT and *Ramp1*-deficient PANC02 tumors for RNA-sequencing (RNA-seq) (Figure 5A). Gene Ontology (GO) enrichment analysis of tumor cells revealed that calcium signaling and growth factor pathways were among the top differentially regulated pathways (Figure 5B). Given that CGRP canonically triggers intracellular calcium influx via the RAMP1/CALCRL complex [31], we observed extensive alterations in calcium-related signaling pathways in *Ramp1*-deficient PDAC cells. Notably, *Cdkn1c*, *Cdkn2a* and *Cdkn2b*, which are canonical downstream effectors of the CGRP-RAMP1/CALCRL axis, were significantly downregulated upon *Ramp1* deficiency, as validated by qPCR (Figure S4A–C). Regarding growth factor pathways, clustering analysis demonstrated a significant downregulation of *Tgfb2*, *Tgfb3*, and *Bmp2* in *Ramp1*-deficient cells (Figure 5C). This transcriptomic reduction was confirmed by qPCR, which verified that *Tgfb2* expression was sharply attenuated in *Ramp1*-deficient PDAC cells, along with *Tgfb3* and *Bmp2* (Figure 5D).

Concurrently, GO enrichment analysis of tumor-infiltrating CD45^+^ immune cells showed significant enrichment of adaptive immune response and type II interferon production pathways in *Ramp1*-deficient tumors (Figure 5E). Clustering of T cell effector and cytotoxicity genes revealed markedly elevated expression of *Tnf*, *Ifng* and *Gzmb* following *Ramp1* deletion (Figure 5F). To further validate the RNA-seq findings, we performed qPCR analysis of representative T-cell effector and cytokine-related genes in CD45^+^ tumor-infiltrating immune cells isolated from g*Scramble* and g*Ramp1* PANC02 tumors. Consistent with the transcriptional changes observed by RNA-seq, *Ifng* expression was significantly increased in tumor-infiltrating immune cells from g*Ramp1* tumors compared with those from g*Scramble* tumors (*p* = 0.0286; Figure 5G). *Tnf*, *Gzmb*, and *Prf1* showed similar trends toward increased expression in the gRamp1 group, although these differences did not reach statistical significance (*p* = 0.2000, 0.1143, and 0.2464, respectively). These qPCR results were therefore broadly consistent with the RNA-seq findings and further supported enhanced IFN-γ-associated immune activity following *Ramp1* deficiency in tumor cells. Additionally, clustering of T cell memory-associated genes showed an increasing trend in *Ramp1*-deficient tumors (Figure S4G). Furthermore, Gene Set Enrichment Analysis (GSEA) further revealed an enhanced IFN-γ response in CD326^+^ CD45^−^ cells within *Ramp1*-deficient tumors (Figure 5H). Collectively, these data indicate that *Ramp1* deficiency in pancreatic cancer cells relieves immunosuppression and promotes T cell effector function and memory potential within the tumor microenvironment.

Based on these findings, we hypothesized that CGRP might promote TGF-β expression in tumor cells via RAMP1/CALCRL, which subsequently acts on infiltrating T cells to blunt their activation and cytotoxic capacity.

### 2.6. CGRP Stimulates Tumor TGF-β2 Expression to Inhibit Effector Function of CD8^+^ T Cells

**Figure 6:**
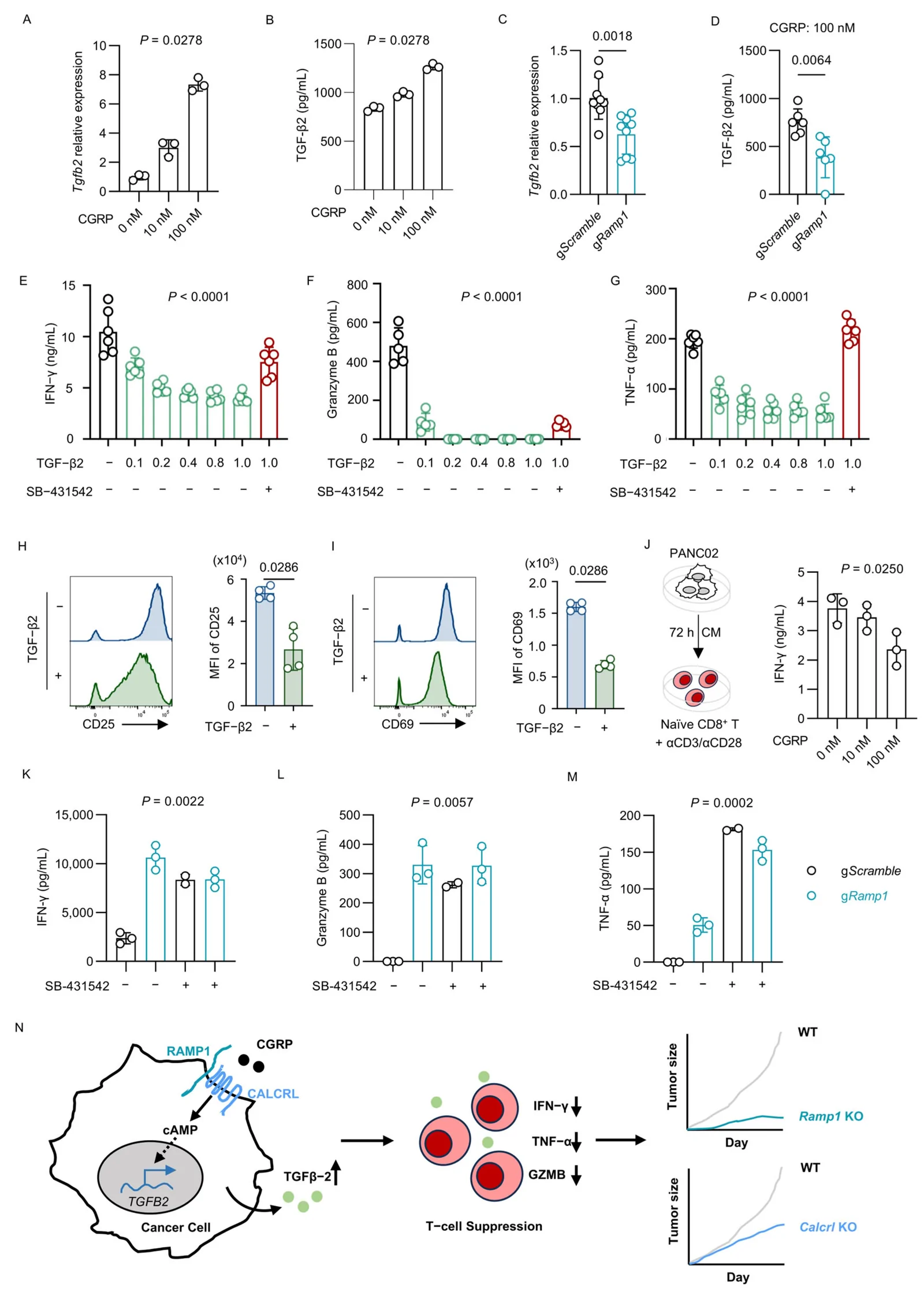
CGRP regulates TGF-β2 secretion by PANC02 cells to suppress CD8^+^ T cell function. (**A**,**B**) PANC02 cells were treated with 0, 10, or 100 nM CGRP for 48 h, followed by analysis of relative *Tgfb2* mRNA expression (**A**) and secreted TGF-β2 concentrations (**B**). Experiments were performed using PANC02 cells from three different passages, representing three independent biological replicates (*n* = 3). (**C**,**D**) Comparison of relative Tgfb2 mRNA expression (**C**) and secreted TGF-β2 concentrations (**D**) between gScramble and Ramp1-deficient PANC02 cells treated with 100 nM CGRP. Experiments were performed using independently cultured cells from different passages (*n* = 9 independent biological replicates per group). (**E**–**G**) ELISA analysis of IFN-γ (**E**), Granzyme B (**F**), and TNF-α (**G**) in the supernatants of C57BL/6J splenic naïve CD8^+^ T cells activated for 72 h in the presence of the indicated concentrations of TGF-β2, with or without the TGF-β type I receptor kinase inhibitor SB-431542. CD8^+^ T cells isolated from six individual mice were used as independent biological replicates (*n* = 6). In (**F**), one sample in the untreated control group was unavailable because of a technical failure during ELISA processing (*n* = 5 for this condition). (**H**,**I**) Flow cytometric analysis of CD25 (**H**) and CD69 (**I**) expression on naïve CD8^+^ T cells activated for 72 h with or without 5 ng/mL TGF-β2. CD8^+^ T cells isolated from four individual mice were used as independent biological replicates (*n* = 4 per group). (**J**) ELISA analysis of IFN-γ secretion by naïve CD8^+^ T cells following 72 h of activation in conditioned medium (CM) from PANC02 cells treated with 0, 10, or 100 nM CGRP. CD8^+^ T cells isolated from three individual mice were used as independent biological replicates (*n* = 3). (**K**–**M**) ELISA analysis of IFN-γ (**K**), Granzyme B (**L**), and TNF-α (**M**) in the supernatants of C57BL/6J splenic naïve CD8^+^ T cells activated for 72 h in CM derived from gScramble or Ramp1-deficient PANC02 cells, with or without SB-431542. CD8^+^ T cells isolated from three individual mice were used as independent biological replicates (*n* = 3). For the third condition in (**K**–**M**), one biological replicate was unavailable because of a technical issue with the corresponding culture well (*n* = 2 for this condition). (**N**) Schematic summary of the proposed mechanism by which CGRP–RAMP1 signaling in pancreatic cancer cells promotes TGF-β2 production and suppresses CD8^+^ T-cell effector function. In panel (N), arrows indicate the proposed direction of signaling and functional effects, and downward arrows indicate reduced levels of the indicated effector molecules. Each data point in (**A**–**M**) represents an independent biological replicate. Data are presented as mean ± SD. Data in (**A**,**B**) and (**E**–**G**) were analyzed using the Friedman test. Comparisons between g*Scramble* and *Ramp1*-deficient cells in (**C**,**D**) were performed using two-tailed unpaired t-tests with Welch’s correction. Comparisons between the two independent groups in (**H**,**I**) were performed using the two-tailed Mann–Whitney U test. Data in (**J**–**M**) were analyzed using the Kruskal–Wallis test. Exact *p* values are shown in the graphs.

While VIP-stimulated TGF-β1 secretion is known to contribute to PDAC immunosuppression [14], we observed that *Ramp1* deficiency in PANC02 cells reduced *Tgfb2* expression (Figure 5C). We therefore evaluated whether CGRP regulates TGF-β2 via RAMP1. Indeed, CGRP administration markedly enhanced both *Tgfb2* transcription and TGF-β2 protein secretion in PANC02 cells (Figure 6A,B). Conversely, upon CGRP stimulation, *Ramp1*-deficient PANC02 cells exhibited profoundly lower *Tgfb2* mRNA and secreted TGF-β2 levels, indicating that CGRP acts through RAMP1 to govern TGF-β2 expression (Figure 6C,D).

To dissect the direct effects of TGF-β2 on CD8^+^ T-cell effector function, we treated naïve T cells with recombinant TGF-β2. As expected, TGF-β2 significantly dampened IFN-γ, TNF-α and Granzyme B production in CD8^+^ T cells, which was effectively reversed by the TGF-β receptor inhibitor SB-431542 [41,42] (Figure 6E–G). Moreover, TGF-β2 markedly downregulated the activation markers CD25 and CD69 expression on CD8^+^ T cells, validating its potent T-cell-suppressive capability (Figure 6H–I).

Given that PANC02 cells secrete more TGF-β2 upon CGRP stimulation (Figure 6B), and that TGF-β2 potently suppresses CD8^+^ T cell effector function, we next sought to determine whether PANC02 primarily suppresses CD8^+^ T cells through paracrine TGF-β2 signaling in the tumor microenvironment. Culturing CD8^+^ T cells with supernatants from CGRP-pretreated WT PANC02 cells profoundly blocked IFN-γ secretion (Figure 6J). Regarding whether CGRP stimulates PANC02 cells via RAMP1 to suppress T cells, supernatants from CGRP-primed WT PANC02 cells severely impaired the production of IFN-γ, Granzyme B, and TNF-α in CD8^+^ T cells, *Ramp1* deletion in PANC02 cells largely relieved this inhibition and restored T-cell effector functions (Figure 6K–M). Notably, addition of the TGF-β receptor inhibitor SB-431542 to T cells treated with WT supernatant completely abrogated the suppression, restoring cytokine and cytotoxin levels to those comparable with the *Ramp1*-deficient group (Figure 6K–M). Collectively, these results indicate that CGRP promotes RAMP1-mediated TGF-β2 production by PDAC cells, which in turn effectively suppresses CD8^+^ T-cell cytotoxic function.

In conclusion, our study uncovers a pivotal role for the nociceptive neuropeptide CGRP in PDAC progression. Specifically, activation of the CGRP-RAMP1/CALCRL axis drives TGF-β2 production within the tumor microenvironment, which strongly suppresses the activation and cytotoxic effector molecule secretion of CD8^+^ T cells, ultimately facilitating tumor progression (Figure 6N).

## 3. Discussion

PDAC remains a formidable therapeutic challenge due to its deeply entrenched, immunosuppressive TME [2,3,43,44,45,46]. While conventional strategies have primarily focused on targeting stromal components or intrinsic immune checkpoints, recent advances in tumor neuroscience have revealed that peripheral nerves actively infiltrate and rewire the tumor terrain to drive malignancy [5,30,47,48]. Here, we investigate this from a neuro-tumor-immunology perspective. Notably, *Ramp1* or *Calcrl* ablation in PDAC tumor cells significantly restricted tumor growth in immunocompetent hosts, but exhibited no effect on tumor kinetics in immunodeficient SCID mice, demonstrating that this neuro-tumor interaction is functionally executed via host adaptive immunity. Mechanistically, we identified a novel signaling cascade wherein CGRP acts on PDAC cells via the RAMP1/CALCRL complex to upregulate TGF-β2 secretion, which subsequently blunts CD8^+^ T-cell activation to facilitate tumor progression.

A previous study has established a neural-stromal circuit in PDAC where sensory nerve-derived CGRP acts on the RAMP1/CALCRL complex expressed by cancer-associated fibroblasts (CAFs), which subsequently suppresses their IL-15 secretion to impair NK cell-mediated innate immunity [19]. Our study uncovers a distinct neuro-immune paradigm by demonstrating that CGRP can directly engage the malignant epithelial compartment itself. By establishing that PDAC tumor cells abundantly express functional RAMP1 and CALCRL receptors, our work bypasses the traditional requirement for stromal intermediates. This direct engagement empowers tumor cells to act as a primary, cell-autonomous relay station that actively translates nociceptive neural inputs into localized adaptive immunosuppression. Consequently, this shifts the focus of PDAC neuro-immunology from indirect stromal modulation to a direct, tumor-intrinsic mechanism that paralyzes CD8^+^ T cell functionality.

The poor response of PDAC to immune checkpoint blockade (ICB) is largely attributable to its immunologically “cold” microenvironment, characterized by insufficient CD8^+^ T cell infiltration and impaired cytotoxicity [3,44,49,50]. Dense stroma and aberrant vasculature physically restrict T cell entry [11,23], while infiltrating T cells often exhibit an exhausted phenotype with elevated inhibitory receptors and dysfunction. Our results, together with recent studies, show that blocking CGRP–CALCRL/RAMP1 axes on tumor cells enhances T cell infiltration and effector function while reducing PD-1 expression. Moreover, PDAC tumors harbor significantly fewer memory T cells than normal tissues [45], and we found that *Ramp1*-deficiency in PANC02 cells conferred resistance to tumor rechallenge in tumor-free mice, accompanied by induction of T cell memory-related genes. Collectively, our findings indicate that blockade of CGRP–CALCRL/RAMP1 axes enhances T cell anti-tumor immunity by augmenting infiltration, effector function, and memory formation, while modestly alleviating exhaustion, which is the rationale for combining this strategy with ICB therapy.

We acknowledge several limitations in this study. First, our functional investigations relied primarily on subcutaneous tumor models rather than orthotopic or spontaneous PDAC systems. Subcutaneous grafts frequently exhibit distinct spatial architectures, with peripheral nerve infiltration density and immune cell composition that may not fully mirror the richly innervated and highly fibrotic native pancreatic microenvironment. Consequently, formal validation utilizing orthotopic implantation will be required to fully capture the physiological relevance of this neuro-immune reflex in situ. Second, while our data firmly position TGF-β2 as a functionally dominant mediator downstream of CGRP–RAMP1/CALCRL activation, the exact intracellular signaling cascades and transcriptional machinery governing CGRP-induced TGF-β2 upregulation in PDAC cells remain to be fully elucidated. Future biochemical profiling will be essential to map the precise molecular gap between CGRP receptor engagement and active *Tgfb2* transcription. Third, the study was not designed or statistically powered for a direct comparison of sex effects. Although the main tumor-growth phenotype was reproduced in an independent male cohort, sex-dependent differences in effect magnitude cannot be excluded. Fourth, CRISPR off-target editing was not experimentally assessed by targeted sequencing or whole-genome approaches, and CALCRL protein loss could not be independently confirmed because the tested antibodies lacked sufficient specificity. Finally, we observed a difference in phenotype magnitude between *Ramp1*-deficient PANC02 and *Calcrl*-deficient KPC tumors. This may reflect intrinsic differences between the two PDAC cell lines rather than distinct biological roles of RAMP1 and CALCRL. However, this explanation requires further experimental support. Studies using matched cellular backgrounds, including single- and double-knockout tumor models, will be needed to clarify whether the observed difference is driven by cell-line-specific properties or by differential contributions of the two receptor components.

Taken together, our findings identify the CGRP–RAMP1/CALCRL axis as a critical, targetable vulnerability in PDAC immunotherapy. Clinically, these data provide a solid rationale for drug repurposing strategies using approved CGRP receptor antagonists like Rimegepant. By blocking this axis at its source, such interventions could shut down tumor-derived TGF-β2 secretion and prevent the paracrine paralysis of infiltrating CD8^+^ T cells. Moreover, this pathway aligns perfectly with ongoing therapeutic developments centered around TGF-β2 inhibition, offering a dual-targeting framework to sensitize notoriously cold tumors to immune checkpoint blockade. In summary, actively disrupting neural hijacking, rather than solely relying on classic co-inhibitory blockade, represents a versatile and ready-to-translate platform capable of augmenting current cancer immunotherapies and achieving superior survival outcomes in PDAC.

## 4. Materials and Methods

### 4.1. Cell Culture and Proliferation Assay

Murine KPC cells (OriCell, M1-101) were kindly provided by Prof. Qi Zhang, Zhejiang University School of Medicine, China, and were originally obtained from OriCell/Cyagen Biosciences, Suzhou, China (Cat. No. M1-101). PANC02 (Procell, Wuhan, China; Cat. No.CL-0736) pancreatic cancer cells, human PANC-1 (Procell, Wuhan, China; Cat. No.CL-0184) cells, and HEK293T (Procell, Wuhan, China; Cat. No.CL-0005) cells were used. Cell lines were authenticated, routinely tested for mycoplasma contamination, and maintained within a defined passage range. HEK293T, KPC and PANC02 cells were cultured in high-glucose DMEM (MACGENE, Beijing, China; Cat. No.CM10013), and PANC-1 cells in RPMI-1640 (MACGENE, Beijing, China; CM10040), supplemented with 10% fetal bovine serum (ExCell, Suzhou, China; Cat. No.FSD500) and 1% penicillin–streptomycin (MACGENE, CC004). Cells were maintained at 37 °C with 5% CO_2_ and passaged at 80–90% confluence using 0.25% trypsin–EDTA (MACGENE, Beijing, China; Cat. No.CC012). For proliferation assays, cells were seeded in 96-well plates in quadruplicate. Viability was measured from 0 to 72 h using CCK-8 reagent (Beyotime, Shanghai, China; Cat. No.C0038). Absorbance was recorded at 450 nm with 630 nm as the reference; blank-subtracted values were used to generate growth curves. For cell-based assays, replicate wells within a single experiment were treated as technical replicates and averaged; independently cultured cells from different passages constituted biological replicates, as specified in the corresponding figure legends.

### 4.2. Animals

Six- to eight-week-old C57BL/6J and severe combined immunodeficient (SCID) mice, weighing approximately 18–20 g, were purchased from Beijing Vital River Laboratory Animal Technology Co., Ltd. (Beijing, China). Female mice were used for all primary in vivo experiments unless otherwise indicated. We performed an independent validation experiment using male C57BL/6J mice (Figure S1B). Female mice were used consistently in the primary cohorts to maintain a uniform sex background within each experiment and reduce sex-related biological variability; the male cohort was intended as an independent validation of the principal tumor-growth phenotype rather than as a powered comparison between sexes.

All mice were housed under specific pathogen-free conditions at 23 ± 2 °C and 40–60% relative humidity on a 12 h light/dark cycle, with ad libitum access to food and water. Five mice were housed per cage with corncob bedding mixed with wood shavings. Mice were allowed to acclimatize to the animal facility for 7 days before the initiation of experiments. After individual identification by ear numbering, body weights were recorded. Mice were stratified according to body weight and then randomly allocated to experimental groups using a random number table to minimize baseline differences in body weight between groups. Animals were randomly assigned to cages, and cage positions were arranged without systematic preference to minimize potential positional bias. All animal procedures were approved by the Institutional Animal Care and Use Committee (IACUC) of the Laboratory Animal Center of Military Medical Sciences (approval no. IACUC-DWZX-2026-522; approval date: 4 March 2026) and were performed in accordance with the relevant institutional guidelines.

Sample sizes were not determined by formal a priori power calculations. For the PANC02 C57BL/6J tumor experiment shown in Figure 3A–D, *n* = 20 mice per group was selected based on preliminary variability, the high frequency of tumor non-engraftment observed in pilot work, and the need to retain sufficient tumor-free mice for the planned rechallenge experiment and survival analysis. For the rechallenge experiment (Figure 3E,F), five tumor-free mice and five age-matched naïve controls were used. For the PANC02 SCID experiment and the KPC C57BL/6J/SCID experiments (Figure 3G–J), *n* = 10 mice per group was selected based on pilot observations and commonly used cohort sizes for these models.

### 4.3. RNA Extraction and Quantitative PCR

Total RNA was extracted with TRIzol (Sigma-Aldrich, T9424) and quantified by spectrophotometry. Qualified RNA was reverse-transcribed using the PrimeScript RT system (TaKaRa, RR036A), and quantitative PCR was performed with SYBR Green master mix (Vazyme, Q712-02) and gene-specific primers (synthesized by Tsingke Biotechnology Co., Ltd.). Expression of *Calcrl*, *Ramp1*, *Ramp2*, *Ramp3*, and other nociceptive signaling receptor genes was evaluated.

### 4.4. CGRP Collection and Enzyme-Linked Immunosorbent Assay (ELISA)

Tumors were washed with ice-cold PBS, weighed, minced, and homogenized in four volumes (*w*/*v*) of 2 N acetic acid (30 s on/5 s off, five cycles). Homogenates were heated at 95 °C for 10 min and centrifuged at 15,000 rpm for 5 min at 4 °C. Supernatants were passed through 50 kDa MWCO filters (MilliporeSigma, Burlington, MA, USA; Cat. No.UFC8050) at 20,000× *g* for 30 min at 4 °C, concentrated, and reconstituted in 150 µL ELISA buffer. CGRP was quantified in duplicate using a commercial ELISA kit (Cayman Chemical, Ann Arbor, MI, USA; Cat. No. A07000). Standards (A06482), controls (A10482), samples, and tracer antibody (A04482) were incubated at 4 °C for 16–20 h. After washing, Ellman’s reagent (A09000) was added, and absorbance was measured at 405 nm. Concentrations were calculated from the standard curve.

### 4.5. Generation of Ramp1-Knockout PANC02 Cells

Stable *Ramp1*-knockout PANC02 cells were generated using a lentiviral CRISPR/Cas9 system. sgRNAs targeting mouse *Ramp1* were cloned into LentiCRISPRv2 (Addgene, 52961) and packaged in HEK293T cells with psPAX2 (Addgene, Watertown, MA, USA; plasmid #63586) and VSV-G (Addgene, Watertown, MA, USA; plasmid #8454). PANC02 cells were transduced in the presence of polybrene (Beyotime Biotechnology, Shanghai, China; Cat. No.C0351) and selected with puromycin (GPC, Beijing, China; Cat. No.AK058). Single-cell clones were isolated by limiting dilution and validated by Sanger sequencing. The targets of *Ramp1* sgRNAs were designed in silico using CHOPCHOP (https://chopchop.cbu.uib.no/, accessed on 30 July 2025) prioritizing high-specificity guides with minimal predicted off-target sites in the murine genome. The exact *Ramp1* target sites are provided in the table below (Table 1). Potential off-target effects of the sgRNA target sequences were assessed using Cas-OFFinder (http://www.rgenome.net/cas-offinder/, accessed on 20 April 2026), and no predicted off-target sites were identified under the specified search parameters. To facilitate cloning of the annealed oligonucleotides into the Esp3I-digested LentiCRISPRv2 vector, appropriate overhang sequences were added to both ends of the sgRNA target sequence. Specifically, CACCG was added to the 5′ end of the forward oligonucleotide, whereas AAAC was added to the 5′ end of the reverse-complementary oligonucleotide, together with an additional C at its 3′ end. For example, for the target sequence 5′-GAAGACGCTATGGTGTGACT-3′, the corresponding oligonucleotides were designed as 5′-CACCGGAAGACGCTATGGTGTGACT-3′ and 5′-AAACAGTCACACCATAGCGTCTTCC-3′. The complementary oligonucleotides were annealed and subsequently inserted into the Esp3I cloning site of LentiCRISPRv2.

### 4.6. Generation of Calcrl-Knockout KPC Cells

*Calcrl*-knockout KPC cells were generated by electroporation of CRISPR/Cas9 ribonucleoprotein complexes. The Calcrl sgRNA was selected using the same in silico specificity criteria; its exact sequence and target site are provided in the table below. The Calcrl-targeting sgRNA was synthesized by GenScript (Nanjing, China) based on the target sequence provided by us, as shown in Table 2. Potential off-target effects of the sgRNA target sequences were assessed using Cas-OFFinder (http://www.rgenome.net/cas-offinder/, accessed on 20 April 2026), and no predicted off-target sites were identified under the specified search parameters. A chemically synthesized sgRNA targeting mouse *Calcrl* (synthesized by GenScript) was complexed with recombinant Cas9 protein (Gibco, Thermo Fisher Scientific, Grand Island, NY, USA; Cat. No.A36499) and delivered using a Lonza 4D-Nucleofector and the appropriate nucleofection kit (Lonza, Cologne, Germany; Cat. No.V4XC-2032). The target-specific sequence was linked to a universal tracrRNA scaffold to generate the complete sgRNA. After recovery, single-cell clones were obtained by limiting dilution and screened by genomic PCR and Sanger sequencing.

**Table 2:** The target DNA sequences of the *Calcrl* gene.

sgRNA Name	Target Position	Target Sequence (5′– 3′)
*Calcrl*-sg#1	Chr2, 84375293	GGTTCTCTTGCCTCTTAATA
*Calcrl*-sg#2	Chr2, 84370458	GCTGGAATGACGTTGCAGCA
*Calcrl*-sg#3	Chr2, 84373252	GACCCCATTCAACAAGCAGA
*Calcrl*-sg#4	Chr2, 84370494	GCCTTTACTGCAATAGGACC
*Calcrl*-sg#5	Chr2, 84348223	CGGAGAAGCAGCACTTGATG
*Calcrl*-sg#6	Chr2, 84351216	TGTAACAATCATCCACCTCA
*Calcrl*-sg#7	Chr2, 84370493	CCTTTACTGCAATAGGACCT

### 4.7. Genomic DNA Extraction and Sanger Sequencing

Genomic DNA was isolated from candidate knockout clones and wild-type controls using a commercial kit (Wayen Biotechnologies (Shanghai), Inc., Shanghai, China; Cat. No.HW0024). CRISPR target regions were amplified and subjected to Sanger sequencing (by Beijing Tsingke Biotech Co., Ltd., Beijing, China). Traces were compared with wild-type controls to identify insertions, deletions, and frameshift mutations. No targeted sequencing of predicted off-target loci or whole-genome off-target analysis was performed. The use of multiple independent *Ramp1* knockout clones reduced, but did not eliminate, the possibility that off-target editing contributed to the observed phenotype.

### 4.8. Subcutaneous Tumor Models

Subcutaneous tumors were established in C57BL/6J and SCID mice by injecting 1 × 10^6^ wild-type or knockout PANC02/KPC cells in 100 µL into the right dorsal flank. For each experimental cohort, systematic longitudinal tumor measurements were initiated when tumors had generally reached an approximate volume range of 30–80 mm^3^, at which point caliper-based measurements could be performed with sufficient reproducibility. Thereafter, tumor length and width, together with body weight, were measured every 3 days. Tumor volume was calculated as 0.5 × length × width^2^.

### 4.9. Flow Cytometric Analysis of Tumor Immune Microenvironment

Fresh tumors were minced and digested using a tumor dissociation kit (Miltenyi Biotec, Bergisch Gladbach, Germany; Cat. No.130-096-730) and DNase I (Yuanye Bio-Technology, Shanghai, China; Cat. No.R21951). Single-cell suspensions were filtered, treated with red blood cell lysis buffer (Gibco, Grand Island, NY, USA; Cat. No.A1049201), and resuspended in FACS buffer. After Fc blockade (BioLegend, San Diego, CA, USA; Cat. No.101302), cells were stained with a viability dye (Thermo Fisher, Waltham, MA, USA; Cat. No.65-0865-18) and fluorochrome-conjugated surface antibodies, followed by intracellular or intranuclear staining using the Cytofix/Cytoperm Soln Kit (BD Biosciences, San Jose, CA, USA; Cat. No.554714) or Foxp3/Transcription Factor Staining Buffer Set (eBioscience, San Diego, CA, USA; Cat. No.00-5523-00). The antibodies used for flow cytometry are listed in Table 3. Data were acquired by flow cytometry and analyzed in FlowJo v10 software (FlowJo LLC, Ashland, OR, USA). to determine the frequencies and absolute numbers of tumor-infiltrating immune-cell subsets.

Gating was performed sequentially to identify lymphocytes by FSC/SSC, singlets, live cells, CD45^+^ leukocytes, and CD4^+^ or CD8^+^ T-cell subsets, followed by analysis of the indicated cytokine and cytotoxic-marker gates. Single-stained controls were used for fluorescence compensation, and fluorescence-minus-one controls were used for Granzyme B, IFN-γ, and TNF-α gates. Approximately 1 × 10^5^–1 × 10^6^ total events were acquired per sample; for cytokine analyses, approximately 1 × 10^4^ events were acquired within the CD4^+^ or CD8^+^ T-cell gates. Cell viability was assessed by trypan-blue exclusion before staining and exceeded 60% in all analyzed samples; downstream analyses were restricted to live-cell gates. When individual Ramp1-deficient tumors were too small to yield sufficient T cells, 2–3 tumors were occasionally pooled, and each resulting pool was treated as a single analytical sample.

**Table 3:** Flow cytometric Antibody Clone Information.

Antibody Name	Catalog No.	Clones	Brand	Dilution
Brilliant Violet 605 anti-mouse CD8a	100743	53-6.7	BioLegend	1:200
FITC anti-mouse CD8a	100706	53-6.7	BioLegend	1:200
Brilliant Violet 421 anti-mouse CD4	100438	GK1.5	BioLegend	1:200
Alexa Fluor 700 anti-mouse CD45	103128	30-F11	BioLegend	1:200
APC anti-mouse IFN-gamma	505810	XMG1.2	BioLegend	1:200
Brilliant Violet 510 anti-mouse CD45	103138	30-F11	BioLegend	1:200
PE anti-mouse TNF-alpha Antibody	506306	MP6-XT22	BioLegend	1:200
FITC anti-human/mouse Granzyme B	515403	GB11	BioLegend	1:200
PE anti-mouse FOXP3 Recombinant	118903	QA20A67	BioLegend	1:200
Brilliant Violet 605 anti-mouse CD25	102035	PC61	BioLegend	1:200
FITC anti-mouse CD69	104505	H1.2F3	BioLegend	1:200
CD326 (EpCAM) Monoclonal Antibody (G8.8), APC	17-5791-82	G8.8	Thermo Fisher	1:200
FITC anti-mouse CD279 (PD-1)	135213	29F.1A12	BioLegend	1:200
CD366 (TIM3) Monoclonal Antibody (RMT3-23), PE-Cyanine7	25-5870-82	RMT3-23	Thermo Fisher	1:200
PE/Cyanine7 anti-mouse NK-1.1 Antibody	108714	PK136	BioLegend	1:200

### 4.10. Preparation of PANC02 Cell-Conditioned Medium and CGRP Stimulation

PANC02 cells, including g*Scramble* control cells and gene knockout cells, were cultured under standard conditions. When cells reached the appropriate confluence, the culture medium was replaced with fresh medium, followed by treatment with CGRP (100 nM) or vehicle control. After 72 h of incubation, the culture supernatants were collected and centrifuged to remove cellular debris. The resulting conditioned medium (CM) was collected and used for subsequent CD8^+^ T cell co-culture experiments. Before application to CD8^+^ T cells, the conditioned medium was normalized to the producer-cell number and total protein concentration to reduce confounding from differences in cell density or viability.

### 4.11. Isolation and Activation of Mouse Splenic CD8^+^ T Cells and Culture Assay

Mouse spleens were collected and mechanically dissociated into single-cell suspensions. Naïve CD8^+^ T cells were purified using a magnetic bead-based isolation kit (Miltenyi, Bergisch Gladbach, Germany; Cat. No.130-096-543) according to the manufacturer’s instructions. After isolation, the naïve CD8^+^ T cells were resuspended and seeded onto plates pre-coated with anti-mouse CD3 antibody (BioLegend, San Diego, CA, USA; Cat. No.100359), and anti-mouse CD28 antibody (BioLegend, San Diego, CA, USA; Cat. No.102121). These T cells were then cultured under two separate experimental settings: (1) in the presence of graded concentrations of recombinant TGF-β2 and/or the TGF-β receptor inhibitor SB-431542 (as indicated); (2) in the presence of conditioned media collected from PANC02 cells that had been cultured for 72 h with or without CGRP stimulation. After 72 h of culture under either condition, the supernatants were harvested for subsequent cytokine analysis.

### 4.12. ELISA of T Cell Cytokines

The collected supernatants from the CD8^+^ T cell co-culture system were analyzed using enzyme-linked immunosorbent assay (ELISA) according to the manufacturer’s instructions. The levels of immune-related cytokines were quantified to evaluate the effects of PANC02-derived soluble factors on CD8^+^ T cell functional responses. ELISA kits used in this study included mouse IFN-γ ELISA kit (BioLegend, San Diego, CA, USA; Cat. No.430804), mouse TNF-α ELISA kit (BioLegend, San Diego, CA, USA; Cat. No.430904), and mouse Granzyme B ELISA kit (R&D Systems, Minneapolis, MN, USA; Cat. No. 88-8022-88).

### 4.13. Statistical Analysis and Data Visualization

Statistical analyses were performed using GraphPad Prism 9.0 (GraphPad Software, San Diego, CA, USA). Data are presented as mean ± SD unless otherwise indicated. Statistical tests were selected according to the experimental design, sample size, and observed distributional characteristics, and the exact test used for each analysis is specified in the corresponding figure legend. Comparisons between two independent groups were performed using a two-tailed unpaired t test (with Welch’s correction where indicated) or the Mann–Whitney U test. Multi-group data were analyzed using the Friedman test, Kruskal–Wallis test, or ANOVA as appropriate. Formal normality tests were not uniformly performed; non-parametric methods were preferentially used for small-sample datasets or when parametric assumptions were not considered appropriate. Longitudinal tumor- and cell-growth curves were analyzed using two-way repeated-measures ANOVA with the Geisser–Greenhouse correction, followed by Dunnett’s multiple-comparisons test where applicable; the time × experimental-group interaction is reported for the tumor-growth curves. Survival curves were estimated using the Kaplan–Meier method and compared using the log-rank (Mantel–Cox) test. For GO enrichment analyses, multiple-testing correction was performed using the Benjamini–Hochberg procedure. Exact *p* values are reported in the figures where applicable. For data visualization, we used Hiplot [51] (https://hiplot.org, accessed on 27 May 2026), a web-based platform for life-science and biomedical data visualization. Hiplot was used to generate Figure 5B–F from tabular data imported through the platform’s spreadsheet interface.

## Figures and Tables

**Table 1:** The target DNA sequences of the *Ramp1* gene.

sgRNA Name	Target Position	Target Sequence (5′–3′)
*Ramp1*-sg#1	Chr1, 91196873	GAAGACGCTATGGTGTGACT
*Ramp1*-sg#2	Chr1, 91196794	CCTGCCGGGACCCTGACTAT
*Ramp1*-sg#3	Chr1, 91180100	CTGCGGGGCCTCCCGCGGTG
*Ramp1*-sg#4	Chr1, 91196807	TGACTATGGGACTCTCATCC
*Ramp1*-sg#5	Chr1, 91196872	GGAAGACGCTATGGTGTGAC

## Data Availability

The data presented in this study are available within the article and its Supplementary Materials. Due to the large file size (>220 MB), the raw data supporting the findings of this study are available from the corresponding author upon reasonable request.

## Supplementary Materials

The following supporting information can be downloaded at: https://www.mdpi.com/article/10.3390/ijms27209108/s1.

## Abbreviations

CGRP	Calcitonin Gene-Related Peptide
RAMP1	Receptor Activity-Modifying Protein 1
CALCRL	Calcitonin Receptor Like Receptor
TGF-β2	Transforming Growth Factor Beta 2
PDAC	Pancreatic Ductal Adenocarcinoma
CRISPR	Clustered Regularly Interspaced Short Palindromic Repeats
TME	Tumor Microenvironment
PNI	Perineural Invasion
TH	Tyrosine Hydroxylase
vAChT	Vesicular Acetylcholine Transporter
ADRB2	Adrenoceptor Beta 2
VIP	Vasoactive Intestinal Peptide
VPAC2	Vasoactive Intestinal Peptide Receptor 2
SP	Substance P
NK-1R	Neurokinin-1 Receptor
PANC1	PANC-1 (human pancreatic cancer cell line)
PANC02	PANC-02 (mouse pancreatic cancer cell line)
KPC	LSL-KrasG12D/+; LSL-Trp53R172H/+; Pdx1-Cre mouse model
SCID	Severe Combined Immunodeficiency
PAAD	Pancreatic Adenocarcinoma (TCGA disease code)
TISCH	Tumor Immune Single-cell Hub
CCK-8	Cell Counting Kit-8
GO	Gene Ontology
GSEA	Gene Set Enrichment Analysis
IFN	Interferon
TNF	Tumor Necrosis Factor
HEK293T	Human Embryonic Kidney 293T cells
qPCR	Quantitative Polymerase Chain Reaction
RNP	Ribonucleoprotein
