# Supplementary material for: CGRP–RAMP1/CALCRL Signaling Drives TGF-β2-Mediated Immune Evasion and Pancreatic Cancer Progression
Source: Int J Mol Sci. 2026 Oct 14;27(20):9108. doi: 10.3390/ijms27209108 (PMC13705189; doi:10.3390/ijms27209108)
Supplement: Supplementary file 1 [file ijms-27-09108-s001.zip › Supplementary Meterials/Suppl Materials.pdf]

Supplementary Materials

qPCR primer sequences

Table S1. Primer sequences used for quantitative PCR

Gene	Forward primer (5′–3′)	Reverse primer (5′–3′)
CALCRL	TCCTGAGGACTCAATTCAGTTGG	CTGTTGCAGTAAACGCCTTCT
RAMP1	CCAGGAGGCTAACTACGGTG	GGGACCACGATGAAGGGGTA
RAMP2	TCCTATCGAAAAGGATTGGTGC	GTTGGCAAAGTGGATCTGGTG
RAMP3	GGGAAGGCTTTCGCAGACAT	TAGCAGCCCACGACATTGG
SSTR1	GCGCCATCCTGATCTCTTTCA	AACGTGGAGGTGACTAGGAAG
SSTR3	AGAACCTGAGAATGCCTCCTC	GCCGCAGGACCACATAGATG
SSTR4	GCATGGTCGCTATCCAGTG	GCGAAGGATCACGAAGATGAC
GALR1	ATCTGCTTCTGCTATGCCAAG	CAGTGGGCGGTGATTCTGA
GALR2	GCCCTACCTGAGCTACTACC	GATGAGGATCATGCGTGTCAC

GALR3	GTGGCTGACCTCTGCTTCAT	AGACAGCAGCCAGCGTAAAG
TACR1 (NK1R)	GCTGCCCTTCCACATCTTCT	CCCAGACGGAACCTGTCATT
Calcrl	ATCTCAGCAGAGTCGGAAGAA	CAGGTCCTATTGCAGTAAAGGC
Ramp1	GAGACTATTGGGAAGACGCTATG	CTCCTCCAGACCACCAGTG
Ramp2	GGAGTCCCTGAACCAATCTCT	GCAACTCTTGTACTCATACCAGC
Ramp3	GTGAGTGTGCCCAGGTATGC	CGACAGGTTGCACCACTTC
Sstr1	CAGGGTAGCGCCATTCTCATC	AGCGTGGAAGTGACCAGAAAG
Sstr2	CGCATGGTGTCCATCGTAGT	GGATTGTGAATTGTCTGCCTTGA
Sstr3	TGTTACCTATCCCTCATCCGAG	GAGAGATCAAGATGCCACTGAC
Sstr4	CAGCGGGCATGGTCACTATC	CCGTCCACGCTAAGCACTG
Sstr5	TTCATCACTTACACGTCTGTGC	AGTCACCTTGCGTTCTGAGC
Galr1	GAACTGGCTATGGTGAACCTC	CCAAACACTACCAGCGTAATGA
Galr2	GCGGTCCTCGTACCCCTATT	CACAGGATGAAACACAGGTCG

Galr3	GTTGGGCATGGTGGGTAATG	CACCGCCAAGTTGAGGATGA
Tacr1	CTCCACCAACACTTCTGAGTC	TCACCACTGTATTGAATGCAGC
Tacr2	GGCCTTGAGAGTAACGCAACA	GGCCAGAATGATCCAGATGACT

All primer sequences are presented in the 5′–3′ direction

sgRNA sequences

Table S2. Candidate sgRNA sequences designed for mouse Calcrl for Cas9/RNP electroporation

sgRNA name	Target sequence (5′–3′)	Length (nt)
Calcrl-sg#1	GGTTCTCTTGCCTCTTAATA	20
Calcrl-sg#2	GCTGGAATGACGTTGCAGCA	20
Calcrl-sg#3	GACCCCATTCAACAAGCAGA	20
Calcrl-sg#4	GCCTTTACTGCAATAGGACC	20
Calcrl-sg#5	CGGAGAAGCAGCACTTGATG	20

Calcrl-sg#6	TGTAACAATCATCCACCTCA	20
Calcrl-sg#7	CCTTTACTGCAATAGGACCT	20

Table S3. Oligonucleotides for lentiviral CRISPR targeting of mouse *Ramp1*

Target gene	sgRNA name	Target sequence (5′–3′)	Forward cloning oligo (5′–3′)	Reverse cloning oligo (5′–3′)
Ramp1	*Ramp1*-sg#1	GAAGACGCTATGGTGTGACT	CACCGGAAGACGCTATGGTGTGACT	AAACAGTCACACCATAGCGTCTTCC
Ramp1	*Ramp1*-sg#2	CCTGCCGGGACCCTGACTAT	CACCGCCTGCCGGGACCCTGACTAT	AAACATAGTCAGGGTCCCGGCAGGC
Ramp1	*Ramp1*-sg#3	CTGCGGGGCCTCCCGCGGTG	CACCGCTGCGGGGCCTCCCGCGGTG	AAACCACCGCGGGAGGCCCCGCAGC
Ramp1	*Ramp1*-sg#4	TGACTATGGGACTCTCATCC	CACCGTGACTATGGGACTCTCATCC	AAACGGATGAGAGTCCCATAGTCAC
Ramp1	*Ramp1*-sg#5	GGAAGACGCTATGGTGTGAC	CACCGGGAAGACGCTATGGTGTGAC	AAACGTCACACCATAGCGTCTTCCC

Antibody Catalog Information

Antibody Name	Catalog No.	Brand	Dilution
Brilliant Violet 605 anti-mouse CD8a	100743	BioLegend	1:200
Brilliant Violet 421 anti-mouse CD4	100438	BioLegend	1:200
Alexa Fluor 700 anti-mouse CD45	103128	BioLegend	1:200
APC anti-mouse IFN-gamma	505810	BioLegend	1:200
PE anti-mouse TNF-alpha Antibody	506306	BioLegend	1:200
FITC anti-human/mouse Granzyme B	515403	BioLegend	1:200
PE anti-mouse FOXP3 Recombinant	118903	BioLegend	1:200
Brilliant Violet 605 anti-mouse CD25	102035	BioLegend	1:200
FITC anti-mouse CD69	104505	BioLegend	1:200
PE/Cyanine7 anti-human CD56 (NCAM)	362510	BioLegend	1:200

CD326 (EpCAM) Monoclonal Antibody (G8.8), APC	17-5791-82	Thermo Fisher Scientific	1:200
FITC anti-mouse CD279 (PD-1)	135213	BioLegend	1:200
CD366 (TIM3) Monoclonal Antibody (RMT3-23), PE-Cyanine7	25-5870-82	Thermo Fisher Scientific	1:200
PE/Cyanine7 anti-mouse NK-1.1 Antibody	108714	BioLegend	1:200
