# Supplementary material for: CGRP–RAMP1/CALCRL Signaling Drives TGF-β2-Mediated Immune Evasion and Pancreatic Cancer Progression
Source: Int J Mol Sci. 2026 Oct 14;27(20):9108. doi: 10.3390/ijms27209108 (PMC13705189; doi:10.3390/ijms27209108)
Supplement: Supplementary file 1 [file ijms-27-09108-s001.zip › Supplementary Meterials/Supplementary Figure Legends.pdf]

Figure S1. RAMP1 and CALCRL expression correlates with tumor growth and survival. (A) Western blot analysis of RAMP1 and GAPDH (loading control) protein expression in KPC and PANC02 murine pancreatic cancer cell lines. (B) Tumorigenesis rates in C57BL/6J mice inoculated with wild-type (WT) or *Ramp1*-deficient PANC02 tumors. Gray bars indicate mice that developed detectable tumors, while blue bars indicate tumor-free mice. (C) Tumor weights of WT and *Ramp1*-deficient PANC02 tumors resected from C57BL/6J mice at endpoint (38 days after inoculation); this experiment represents a repeat of the findings shown in Figure 3A. Data are shown as individual points with median and interquartile range. Sample sizes were n = 7 for gScramble and n = 10 for gRamp1. Statistical analysis was performed using a two-tailed Mann-Whitney U test ($U = 0.00$, $P < 0.0001$). (D) Individual tumor growth curves of WT and *Ramp1*-deficient PANC02 tumors subcutaneously implanted into SCID mice. (E) Individual tumor growth curves of WT and *Calcrl*-deficient KPC tumors implanted into syngeneic C57BL/6J mice. (F) Overall survival of C57BL/6J mice bearing WT or *Calcrl*-deficient KPC tumors. (G) Individual tumor growth curves of WT and *Calcrl*-deficient KPC tumors subcutaneously implanted into immunodeficient SCID mice.

Figure S2. Generation of Ramp1-deficient PANC02 and Calcrl-deficient KPC cell lines. (A) Schematic of the CRISPR-Cas9 strategy used to disrupt *Ramp1* in PANC02 cells via the Lenti-V2 system. (B) Sanger sequencing chromatograms confirming *Ramp1* knockout in PANC02 cells, with the targeted guide RNA (gRNA) sequence and protospacer adjacent motif (PAM) indicated. (C) Sequencing peak overlay (or electropherogram) showing the frameshift mutation induced in the *Ramp1* locus in PANC02 cells, marked by red. (D) Schematic of the CRISPR-Cas9 ribonucleoprotein (RNP) nucleofection strategy used to disrupt *Calcrl* in KPC cells. (E) Sanger sequencing chromatograms confirming *Calcrl* knockout in KPC cells (F) Sequencing peak overlay (or electropherogram) demonstrating the frameshift mutation at the *Calcrl* locus in KPC cells.

Figure S3. T cell subtype composition and exhaustion marker expression in PANC02 tumors. (A-C) Flow cytometric analysis of T cell exhaustion markers on tumor-infiltrating $CD8^+$ T lymphocytes. (A) Representative flow cytometry plots showing the gating strategy for $CD8^+$ T cells. (B) Quantification of PD-1^+ and PD-1^+ TIM-3^+ double-positive cells among $CD8^+$ T cells infiltrating wild-type (WT) versus Ramp1-deficient PANC02 tumors. (C) Mean fluorescence intensity (MFI) of PD-1 expression on $CD8^+$ tumor-infiltrating lymphocytes (TILs) in WT versus Ramp1-deficient tumors. (D) Frequency of regulatory T cells (Tregs),

defined as $CD25^{+}Foxp3^{+}$ cells within the $CD4^{+}$ T cell population, in WT and Ramp1-deficient tumors. (E) Complete gating workflow illustrating the sorting strategy for the isolation of PANC02 tumor cells and tumor-infiltrating immune cells. Data are presented as mean ± SEM. Statistical significance was determined using [insert specific test, e.g., unpaired two-tailed Student's t-test or Mann-Whitney U test]; *$p < 0.05$, **$p < 0.01$, ***$p < 0.001$; n.s., not significant. (A) Mean fluorescence intensity of PD-1 on $CD8^{+}$ tumor-infiltrating lymphocytes in gScramble and Ramp1-deficient tumors. (B) Percentage of PD-1^{+} cells among $CD8^{+}$ T cells. (C) Percentage of PD-1^{+}TIM-3^{+} cells among $CD8^{+}$ T cells. (D) Frequency of regulatory T cells, defined as $CD25^{+}$ $Foxp3^{+}$ cells among $CD4^{+}$ T cells. (E) Complete gating workflow illustrating the sorting strategy used to isolate PANC02 tumor cells and tumor-infiltrating immune cells. Data are presented as mean ± SD. Comparisons in (A-D) were performed using Mann-Whitney U test. Exact P values are shown in the graphs.

Figure S4. RNA expression profiles of calcium signaling, BMP pathway, and T cell-related gene signatures in PANC02 tumors. (A-C) Quantitative real-time PCR (qPCR) validation of calcium signaling-related genes *Cdkn1c, Cdkn2a, and Cdkn2b* that were significantly differentially expressed in *Ramp1*-deficient versus wild-type (WT) tumor cells, as identified by RNA-seq. (D-F) qPCR validation of BMP pathway-related genes (*Bmp3*, *Bmp5*, and *Gdf10*) that were significantly altered upon

Ramp1 deficiency based on RNA-seq data from tumor cells. (G) Hierarchical clustering heatmap of T cell activation- and memory-associated gene expression in tumor-infiltrating immune cells from WT versus *Ramp1*-deficient tumors, generated using the Hiplot platform. Rows: genes; columns: samples; color scale: Z-score-normalized expression (blue, low; red, high). Dendrograms indicate gene and sample clustering. *Ramp1*-deficient tumors displayed distinct clustering patterns from WT controls for both activation- and memory-related gene sets. Data are presented as mean ± SD. Comparisons in (A-F) were performed using Mann-Whitney U test. Exact P values are shown in the graphs.
