# Supplementary material for: CGRP–RAMP1/CALCRL Signaling Drives TGF-β2-Mediated Immune Evasion and Pancreatic Cancer Progression
Source: Int J Mol Sci. 2026 Oct 14;27(20):9108. doi: 10.3390/ijms27209108 (PMC13705189; doi:10.3390/ijms27209108)
Supplement: Supplementary file 1 [file ijms-27-09108-s001.zip › Supplementary Meterials/Supplementary Figures.pdf]

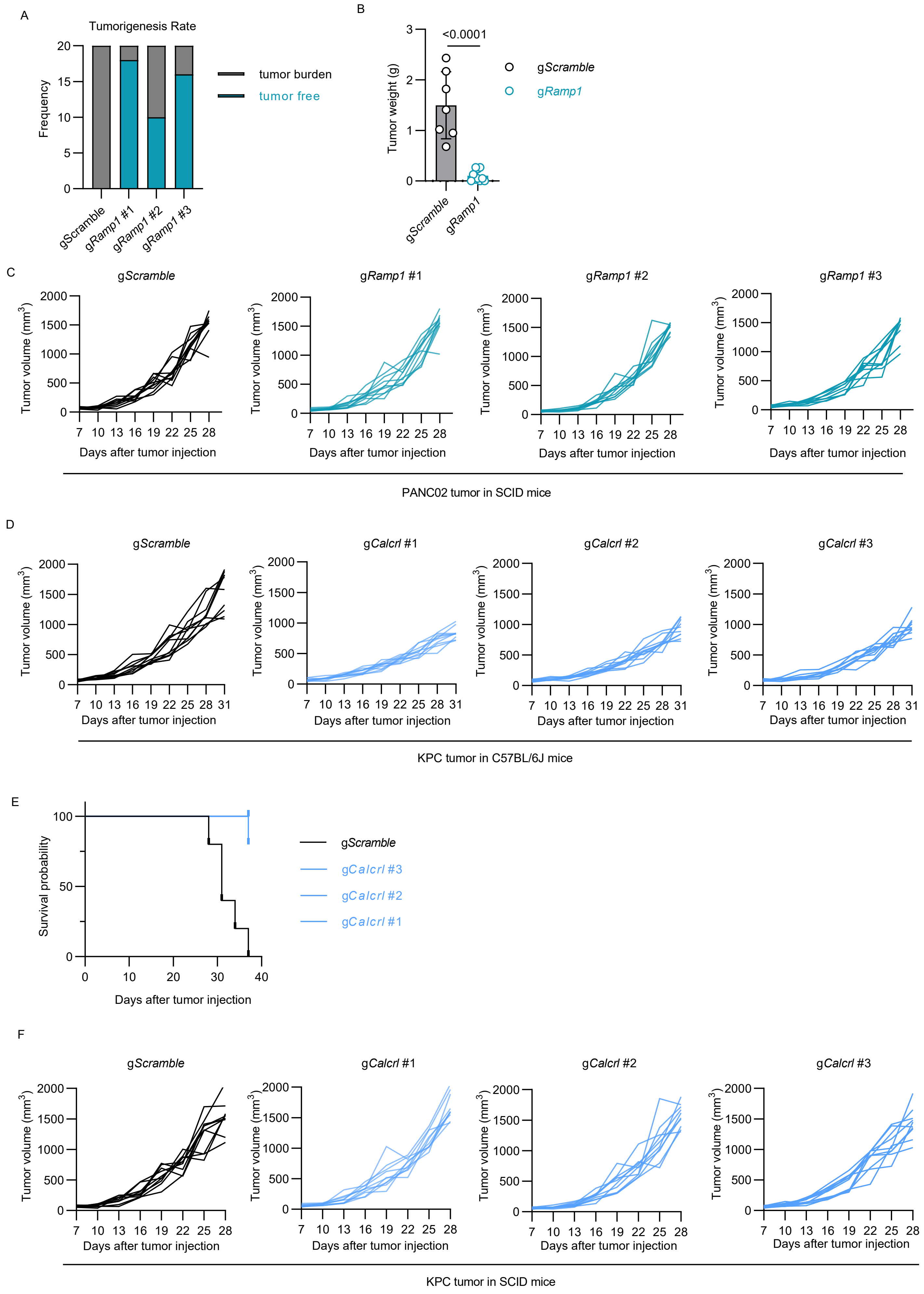

A
Tumorigenesis Rate
Frequency
20
15
10
5
0
gScramble
gRamp1 #1
gRamp1 #2
gRamp1 #3
tumor burden
tumor free
B
Tumor weight (g)
3
2
1
0
<0.0001
gScramble
gRamp1
C
gScramble
gRamp1 #1
gRamp1 #2
gRamp1 #3
Tumor volume (mm3)
2000
1500
1000
500
0
7 10 13 16 19 22 25 28
Days after tumor injection
PANC02 tumor in SCID mice
D
gScramble
gCalcrl #1
gCalcrl #2
gCalcrl #3
Tumor volume (mm3)
7 10 13 16 19 22 25 28 31
Days after tumor injection
KPC tumor in C57BL/6J mice
E
Survival probability
100
50
0
0 10 20 30 40
Days after tumor injection
gScramble
gCalcrl #3
gCalcrl #2
gCalcrl #1
F
gScramble
gCalcrl #1
gCalcrl #2
gCalcrl #3
Tumor volume (mm3)
7 10 13 16 19 22 25 28
Days after tumor injection
KPC tumor in SCID mice

**Figure S1. RAMP1 and CALCRL expression correlates with tumor growth and survival.** (A) Western blot analysis of RAMP1 and GAPDH (loading control) protein expression in KPC and PANC02 murine pancreatic cancer cell lines. (B) Tumorigenesis rates in C57BL/6J mice inoculated with wild-type (WT) or *Ramp1*-deficient PANC02 tumors. Gray bars indicate mice that developed detectable tumors, while blue bars indicate tumor-free mice. (C) Tumor weights of WT and *Ramp1*-deficient PANC02 tumors resected from C57BL/6J mice at endpoint (38 days after inoculation); this experiment represents a repeat of the findings shown in Figure 3A. Data are shown as individual points with median and interquartile range. Sample sizes were $n = 7$ for gScramble and $n = 10$ for gRamp1. Statistical analysis was performed using a two-tailed Mann-Whitney U test ($U = 0.00$, $P < 0.0001$). (D) Individual tumor growth curves of WT and *Ramp1*-deficient PANC02 tumors subcutaneously implanted into SCID mice. (E) Individual tumor growth curves of WT and *Calcrl*-deficient KPC tumors implanted into syngeneic C57BL/6J mice. (F) Overall survival of C57BL/6J mice bearing WT or *Calcrl*-deficient KPC tumors. (G) Individual tumor growth curves of WT and *Calcrl*-deficient KPC tumors subcutaneously implanted into immunodeficient SCID mice.

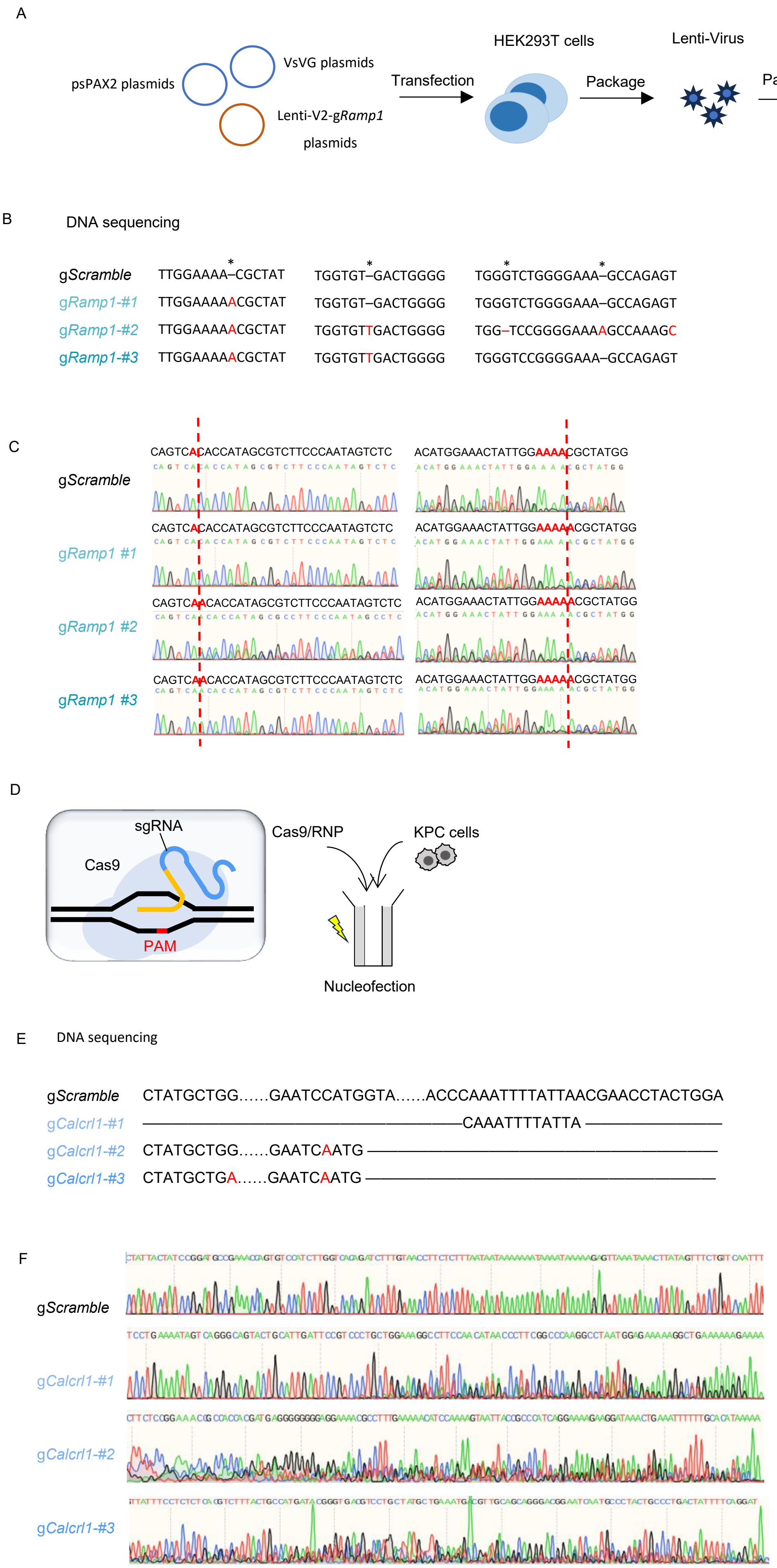
A
psPAX2 plasmids
VsVG plasmids
Lenti-V2-gRamp1 plasmids
Transfection
HEK293T cells
Package
Lenti-Virus
Package
PANC02 cells
B
DNA sequencing
gScramble TTGGAAAA–CGCTAT TGGTGT–GACTGGGG TGGGTCTGGGGAAA–GCCAGAGT
gRamp1-#1 TTGGAAAAACGCTAT TGGTGT–GACTGGGG TGGGTCTGGGGAAA–GCCAGAGT
gRamp1-#2 TTGGAAAAACGCTAT TGGTGTTGACTGGGG TGG–TCCGGGGAAAAGCCAAAGC
gRamp1-#3 TTGGAAAAACGCTAT TGGTGTTGACTGGGG TGGGTCCGGGGAAA–GCCAGAGT
C
gScramble CAGTCACACCATAGCGTCTTCCCAATAGTCTC ACATGGAAACTATTGGAAAACGCTATGG
gRamp1 #1 CAGTCACACCATAGCGTCTTCCCAATAGTCTC ACATGGAAACTATTGGAAAAACGCTATGG
gRamp1 #2 CAGTCAACACCATAGCGTCTTCCCAATAGTCTC ACATGGAAACTATTGGAAAAACGCTATGG
gRamp1 #3 CAGTCAACACCATAGCGTCTTCCCAATAGTCTC ACATGGAAACTATTGGAAAAACGCTATGG
D
sgRNA
Cas9
PAM
Cas9/RNP
KPC cells
Nucleofection
E
DNA sequencing
gScramble CTATGCTGG......GAATCCATGGTA......ACCCAAATTTTATTAACGAACCTACTGGA
gCalcrl1-#1 ——CAAATTTTATTA——
gCalcrl1-#2 CTATGCTGG......GAATCAATG——
gCalcrl1-#3 CTATGCTGA......GAATCAATG——
F
gScramble
gCalcrl1-#1
gCalcrl1-#2
gCalcrl1-#3

**Figure S2. Generation of Ramp1-deficient PANC02 and Calcrl-deficient KPC cell lines**. (A) Schematic of the CRISPR-Cas9 strategy used to disrupt Ramp1 in PANC02 cells via the Lenti-V2 system. (B) Sanger sequencing chromatograms confirming Ramp1 knockout in PANC02 cells, with the targeted guide RNA (gRNA) sequence and protospacer adjacent motif (PAM) indicated. (C) Sequencing peak overlay (or electropherogram) showing the frameshift mutation induced in the Ramp1 locus in PANC02 cells, marked by red. (D) Schematic of the CRISPR-Cas9 ribonucleoprotein (RNP) nucleofection strategy used to disrupt Calcrl in KPC cells. (E) Sanger sequencing chromatograms confirming Calcrl knockout in KPC cells (F) Sequencing peak overlay (or electropherogram) demonstrating the frameshift mutation at the Calcrl locus in KPC cells.

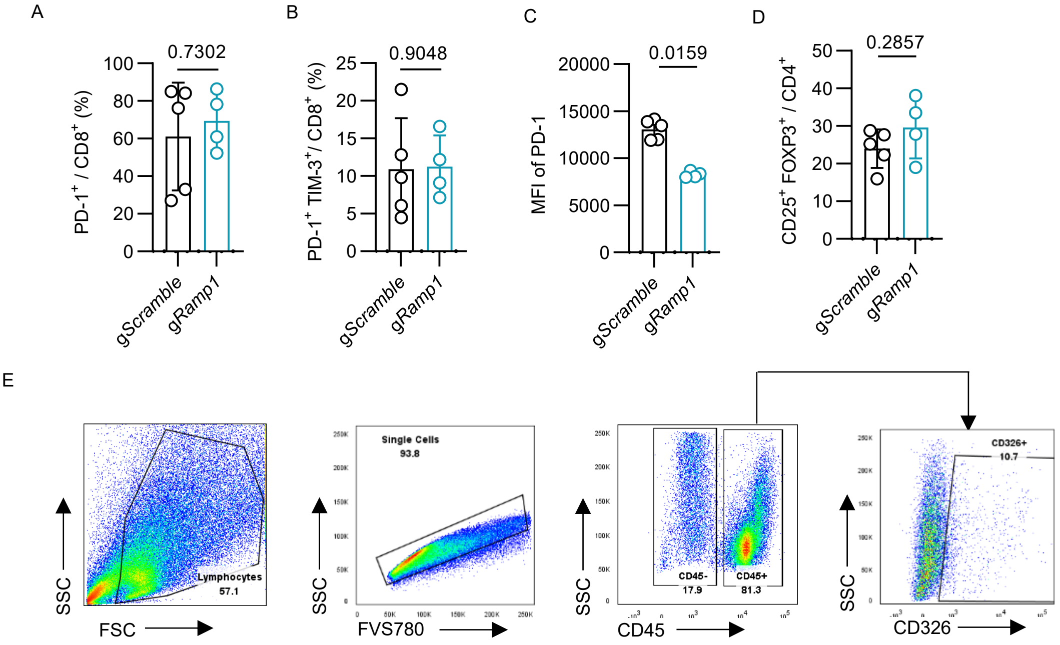


**Figure S3. T cell subtype composition and exhaustion marker expression in PANC02 tumors.** (A-C) Flow cytometric analysis of T cell exhaustion markers on tumor-infiltrating $CD8^+$ T lymphocytes. (A) Representative flow cytometry plots showing the gating strategy for $CD8^+$ T cells. (B) Quantification of $PD\text{-}1^+$ and $PD\text{-}1^+$ $TIM\text{-}3^+$ double-positive cells among $CD8^+$ T cells infiltrating wild-type (WT) versus Ramp1-deficient PANC02 tumors. (C) Mean fluorescence intensity (MFI) of PD-1 expression on $CD8^+$ tumor-infiltrating lymphocytes (TILs) in WT versus Ramp1-deficient tumors. (D) Frequency of regulatory T cells (Tregs), defined as $CD25^+Foxp3^+$ cells within the $CD4^+$ T cell population, in WT and Ramp1-deficient tumors. (E) Complete gating workflow illustrating the sorting strategy for the isolation of PANC02 tumor cells and tumor-infiltrating immune cells. Data are presented as mean ± SEM. Statistical significance was determined using [insert specific test, e.g., unpaired two-tailed Student's t-test or Mann-Whitney U test]; *p < 0.05, **p < 0.01, ***p < 0.001; n.s., not significant. (A) Mean fluorescence intensity of PD-1 on $CD8^+$ tumor-infiltrating lymphocytes in gScramble and Ramp1-deficient tumors. (B) Percentage of $PD\text{-}1^+$ cells among $CD8^+$ T cells. (C) Percentage of $PD\text{-}1^+TIM\text{-}3^+$ cells among $CD8^+$ T cells. (D) Frequency of regulatory T cells, defined as $CD25^+$ $Foxp3^+$ cells among $CD4^+$ T cells. (E) Complete gating workflow illustrating the sorting strategy used to isolate PANC02 tumor cells and tumor-infiltrating immune cells. Data are presented as mean ± SD. Comparisons in (A-D) were performed using Mann-Whitney U test. Exact P values are shown in the graphs.

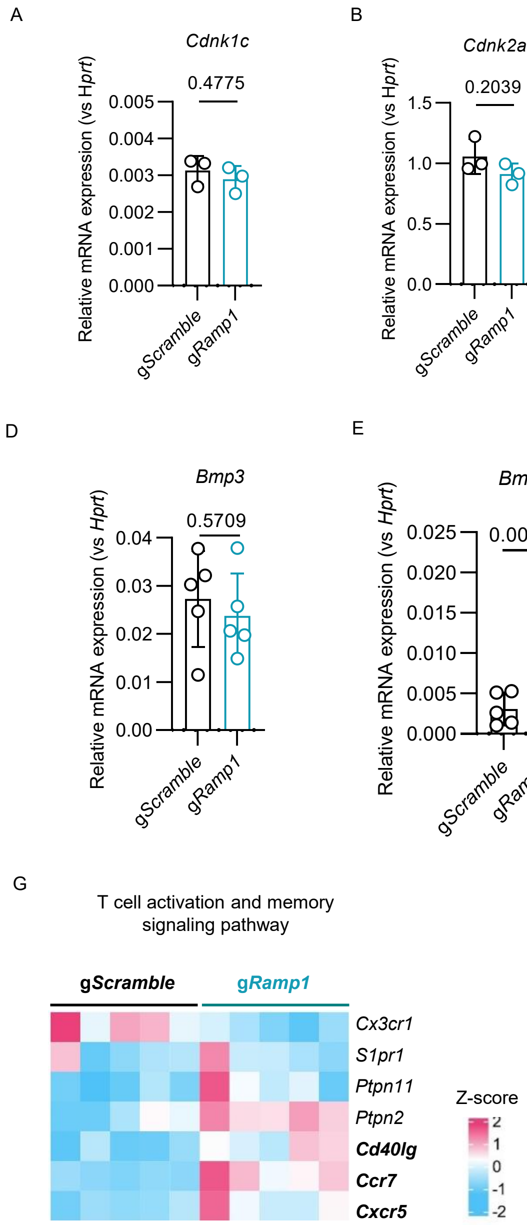
A
Cdnk1c
0.4775
Relative mRNA expression (vs Hprt)
0.005
0.004
0.003
0.002
0.001
0.000
gScramble
gRamp1
B
Cdnk2a
0.2039
Relative mRNA expression (vs Hprt)
1.5
1.0
0.5
0.0
gScramble
gRamp1
C
Cdnk2b
0.0003
Relative mRNA expression (vs Hprt)
0.006
0.004
0.002
0.000
gScramble
gRamp1
D
Bmp3
0.5709
Relative mRNA expression (vs Hprt)
0.04
0.03
0.02
0.01
0.00
gScramble
gRamp1
E
Bmp5
0.0035
Relative mRNA expression (vs Hprt)
0.025
0.020
0.015
0.010
0.005
0.000
gScramble
gRamp1
F
Gdf10
0.1718
Relative mRNA expression (vs Hprt)
0.03
0.02
0.01
0.00
gScramble
gRamp1
G
T cell activation and memory signaling pathway
gScramble
gRamp1
Cx3cr1
S1pr1
Ptpn11
Ptpn2
Cd40lg
Ccr7
Cxcr5
Z-score
2
1
0
-1
-2

**Figure S4. RNA expression profiles of calcium signaling, BMP pathway, and T cell-related gene signatures in PANC02 tumors.** (A-C) Quantitative real-time PCR (qPCR) validation of calcium signaling-related genes Cdkn1c, Cdkn2a, and Cdkn2b that were significantly differentially expressed in Ramp1-deficient versus wild-type (WT) tumor cells, as identified by RNA-seq. (D-F) qPCR validation of BMP pathway-related genes (Bmp3, Bmp5, and Gdf10) that were significantly altered upon Ramp1 deficiency based on RNA-seq data from tumor cells. (G) Hierarchical clustering heatmap of T cell activation- and memory-associated gene expression in tumor-infiltrating immune cells from WT versus Ramp1-deficient tumors, generated using the Hiplot platform. Rows: genes; columns: samples; color scale: Z-score-normalized expression (blue, low; red, high). Dendrograms indicate gene and sample clustering. Ramp1-deficient tumors displayed distinct clustering patterns from WT controls for both activation- and memory-related gene sets. Data are presented as mean ± SD. Comparisons in (A-F) were performed using Mann-Whitney U test. Exact P values are shown in the graphs.
